# Inflammatory bowel diseases, interleukin-6 and interleukin-6 receptor subunit alpha in causal association with cerebral cortical structure: a Mendelian randomization analysis

**DOI:** 10.3389/fimmu.2023.1154746

**Published:** 2023-04-20

**Authors:** Chunlong Liu, Shijie Zhu, Jian Zhang, Kuiwu Ren, Kangkang Li, Jiangtao Yu

**Affiliations:** ^1^ Department of Hepatobiliary and Pancreatic Surgery, Fuyang People’s Hospital, Anhui Medical University, Fuyang, China; ^2^ Department of Occupational and Environmental Health, School of Public Health, Wuhan University, Wuhan, China; ^3^ Department of Neurosurgery, The Seventh Clinical College of China Medical University, Fushun, China; ^4^ Department of Hepatobiliary and Pancreatic Surgery, Fuyang People’s Hospital, Bengbu Medical College, Fuyang, China

**Keywords:** inflammatory bowel disease, Crohn’s disease, ulcerative colitis, brain cortical structure, IL-6, IL-6Rα, Mendelian randomization

## Abstract

**Background:**

Neurological involvement and psychiatric manifestations have been documented in clinical cases of inflammatory bowel disease (IBD); however, the presence of a causal relationship remains elusive. The objective of this study is to investigate the modifications occurring in the cerebral cortex as a result of IBD.

**Methods:**

A compendium of data extracted from a genome-wide association study (GWAS) involving a maximum of 133,380 European subjects. A series of Mendelian random analyses were applied to exclude heterogeneity and pleiotropy, ensuring the stability of the results.

**Results:**

Neither IBDs nor inflammatory cytokines (IL-6/IL-6Rα) were found to have a significant causality with surface area (SA) and thickness (TH) at the global level. At the regional functional brain level, Crohn’s disease (CD) significantly decreased the TH of pars orbitalis (β=-0.003mm, Se=0.001mm, p_ivw_ =4.85×10^-4^). IL-6 was observed to reduce the SA of middle temporal (β=-28.575mm^2^, Se=6.482mm^2^, p_ivw_=1.04×10^-5^) and increase the TH of fusiform (β=0.008mm, Se=0.002mm, p_ivw_=8.86×10^-5^) and pars opercularis (β=0.009mm, Se=0.002mm, p_ivw_=2.34×10^-4^). Furthermore, a causal relationship between IL-6Rα and an increase in the SA of superior frontal (β=21.132mm^2^, Se=5.806mm^2^, p_ivw_=2.73×10^-4^) and the TH of supramarginal (β=0.003mm, Se=0.0002mm, p_ivw_=7.86×10^-37^). All results passed sensitivity analysis and no heterogeneity and pleiotropy were detected.

**Conclusion:**

The correlation between IBD and changes in cerebral cortical structures implies the existence of a gut-brain axis at the organismal level. It is recommended that clinical patients with IBD prioritize long-term management of inflammation, as changes at the organismal level can lead to functional pathologies. Magnetic resonance imaging (MRI) may be considered as an additional screening option for IBD.

## 1 Introduction

Inflammatory bowel disease (IBD) refers to a cluster of intestinal disorders, marked by persistent non-infectious inflammation (1). IBD can be subcategorized into Crohn’s disease (CD), ulcerative colitis (UC), and indeterminate colitis, based on the characteristics of clinical symptoms of inflammation invasion (2). The global incidence and prevalence of IBD have been on a steady rise in recent years, albeit indicating a noteworthy reduction in the fatal disease burden of inflammatory bowel disease (3). However, patients continue to suffer from recurrent symptoms of inflammation and abdominal pain, which tend to negatively impact their overall well-being and emotional state.

The profound interconnection between the gastrointestinal and nervous systems, discovered in the 21st century and named the “gut-brain axis”, is involved in the pathogenesis of IBD. Several population-based cohort studies have exposed a higher incidence of psychiatric disorders, including anxiety and depression, in patients with IBD (4–7). Additionally, patients with IBD are susceptible to developing neurodegenerative diseases such as Parkinson’s disease, dementia, and Alzheimer’s disease, which are secondary to the progression of the disease (8–10). Apart from functional modifications, organismal changes have also been observed. Bao et.al, by using Magnetic Resonance Imaging (MRI), detected significant alterations in grey matter (GM) structure in brain regions of Crohn’s disease patients, which may explain the higher levels of anxiety and depression in those patients (11). Correspondingly, inflammation is the predominant pathological feature in IBD patients. A causal association might exist between inflammation and psychiatric disorders or cerebral cortical structures (12). Interleukin-6 (IL-6) was one of the biomarkers of inflammation in the body (13, 14), and the binding of interleukin-6 to its receptor subunit alpha (IL-6Rα) plays a vital role in the development of IBD (15). Some studies have reported that changes in the levels of interleukin-6 in the body could result in changes in brain structure and lead to neuropsychiatric disorders (16, 17). Dysbiosis of the intestinal microbiota, bacterial product translocation, and the inflammatory soluble factors crossing the intestinal epithelial barrier and the blood-brain barrier (BBB) have been widely accepted as crucial factors in structural and functional changes of the central nervous system (CNS) (18–22).

These mechanisms involve microbial, neural, immune, and endocrine regulation, suggesting that the gut-brain axis may play a vital role in the pathogenesis of intestinal and neurological disorders. However, previous observational studies inevitably had some biases, such as inadequate sample sizes, discrepancies in socio-demographic characteristics, and the pre-existing diseases that may have an impact on brain structure, which diminished the reliability of the conclusion. This study aims to build on the existing research evidence to further explore the gut-brain association at the organismal level and add more frontier research evidence.

Mendelian Randomization (MR) analysis, based on the law of segregation, can surmount the various confounding biases inherent in observational studies and provide a high quality of evidence that approximates that of a randomized controlled study (23, 24). MR studies have been conducted to reveal a causality between IBDs and a number of diseases or risk factors (25–27). In the framework of MR analysis, the summary data of genome-wide association studies (GWAS) on human IBDs and inflammatory cytokines were utilized to predict genetic alterations in global and 34 subregions of cerebral cortical structure, defined in two directions (surface area and thickness). A series of sensitivity analyses was used to exclude heterogeneity and pleiotropy and to confirm the reliability of causal associations. Our study adds new value to the study of the gut-brain axis.

## 2 Method

### 2.1 Exposure data sources for inflammatory bowel disease, Crohn’s disease, and ulcerative colitis

We extracted summary-level data for GWAS of IBD from a meta-analysis of European-ancestry participants. This comprised two components: (1) a newly included GWAS of IBD involving 25,305 individuals (12,160 cases and 13,145 controls) of European ancestry who have not been included in other IBD genome-wide meta-analysis studies to date, and (2) summary-level data of 34,652 individuals (12,882 cases and 21,770 controls) previously published by the IIBDGC consortium (28), which were used for meta-analysis in conjunction with the new GWAS data. The latter data were imputed using a reference panel of 1000 individuals. Participants who consented to participate in the study were diagnosed with IBD using recognized diagnostic criteria of endoscopy, histopathology, and radiology, and were genotyped using the Human Core Exome v12.1. The basic characteristics of the participants were reported by de Lange, K.M., et al. (29). The association summary statistics can be accessed from ftp://ftp.sanger.ac.uk/pub/project/humgen/summary_statistics/human/2016-11-07/. **Supplementary Materials** provided detailed information for the IBD, Crohn’s disease (CD), and ulcerative colitis (UC) population cohorts.

### 2.2 Exposure data sources for interleukin-6 levels and interleukin-6 receptor subunit alpha levels

The summary-level GWAS data of IL-6 levels and IL-6Rα levels were acquired from a genome-wide meta-analysis of 90 cardiovascular-associated proteins from 13 cohorts of European ancestry (30). The cohorts contained a total of 21758 individuals. However, not all proteins passed the cohort quality control process, further information can be found in **Supplementary Materials**.

### 2.3 Outcome data sources: cerebral cortical structure

We applied to the ENIGMA Consortium for GWAS data related to the cerebral cortical structure (31). The study conducted a meta-analysis of GWAS of brain MRI data from 60 cohorts comprising of 51,665 individuals, primarily of European ancestry from various parts of the world. A total of 70 phenotypes were generated, based on 34 brain regions defined by the commonly used Desikan-Killiany atlas, in conjunction with global measures such as total cortical surface area (SA) and mean thickness (TH). GWAS were carried out for each phenotype within each cohort, using additive models. Given that each region has unique genetic influences, the primary GWAS data was adjusted with global measured SA and TH as covariates. The primary GWAS data was balanced with global weighting, to address the differences in SA and TH between regions due to genetic effects, allowing comparability between regions. Furthermore, the data without global weighting was also made available. The present study carried out MR analysis on the primary GWAS data (global-weighted) of cerebral cortex structure.

### 2.4 The selection of genetic instrumental variables

Genetic variants were used as instruments to estimate the causal relationship in MR analysis. Three sets of instrumental variables (IV) were utilized to represent IBD, CD, and UC exposures respectively. Criteria for screening instrumental variables included: (1) the GWAS meta-analysis results showed a high correlation (p<5×10^-8^) between phenotype and single nucleotide polymorphism (SNP). (2) linkage disequilibrium (LD): r^2^<0.001, clumping distance=10000 kb. Weak IVs were generally IVs that were not strongly correlated with exposure factors, which were less effective in explaining genetic variation in exposure factors. The existence of weak IVs leads to an increased deviation of the estimated values from the true values. The F-statistic measures the strength of the IV. The formula for calculating the F-statistic can be expressed as follows: $F=\left(\frac{R^{2}}{1-R^{2}}\right)\left(\frac{n-k-1}{k}\right)$ ; where R(2) represented the extent to which the exposure could be explained by the SNP, n was the sample size and k was the count of SNP. The formula for R(2) can be expressed as: $R^{2}=2\times MAF\times \left(1-MAF\right)\times \beta^{2}$ , where MAF referred to the minor allele frequency, β referred to the effect value of exposure. In the absence of MAF, R2 was calculated using $R^{2}=\frac{\beta^{2}}{\beta^{2}+Se^{2}\times n}$ instead (32), where Se referred to the standard error for effect values of exposure, n represented the sample size. The F-statistic value less than 10 was considered a weak IV. Specific information for instrumental variables and F-statistic values can be viewed in **Supplementary Materials**. Of the inflammatory cytokines, the role of IL-6 and its receptor interactions in the clinical onset and progression of IBDs has been observed and the possible association of inflammatory agents with brain structures suggests a possible mechanism for the gut-brain connection. Two sets of instrumental variables were utilized to represent IL-6 and IL-6Rα respectively, and screening criteria were consistent with IBD. However, due to the rigorous screening criteria, only one instrumental variable for IL-6 was available, which limited the use of multiple analytical methods, potentially leading to bias in the interpretation of results. We appropriately relaxed the screening criteria for instrumental variables of IL-6 (p<5×10^-6^, but F-statistic value >10) while ensuring the interpretability of the results. Mendelian randomization pleiotropy residual sum and outlier (MR PRESSO) will be used to identify instrumental variables (outliers) with significant heterogeneity (outlier test p-value<0.05), which will be removed before MR analysis (33). In order to minimize correlated pleiotropy, we checked the second genotype and phenotype in PhenoScanner (http://www.phenoscanner.medschl.cam.ac.uk/), a database of comprehensive human genotype-phenotype associations, to avoid the potential to avoid potential confounders obscuring the true associations. We deleted SNPs that were associated with a second phenotype that could potentially lead to alterations in cerebral cortical structure, including high body mass index (≥30kg/m^2^), obesity, hyperlipemia, hypertension, neurodegeneration, neuropsychiatric, smoking (current/previous), excessive alcohol consumption(current/previous), to obtain a more confirmed association between IBD, IL-6 and IL-6Rα and cerebral cortical structure.

## 2.5 Two-sample Mendelian randomization analysis

Multiplicative random-effects inverse variance weighting (IVW), weighted median (WM), and MR Egger were performed in MR analysis. The results of multiplicative random-effects IVW, compared with fix-effects IVW, remained robust in the presence of heterogeneity in the selection of instrumental variables, and the parameter estimates were more conservative and more realistic (34). Therefore, we judged the results of IVW as the primary outcome of MR analysis. To make our conclusions more reliable, we required the results of the WM to be significant and the results of WM and MR-Egger were in the same direction as the IVW when the results of IVW were significant, but the WM itself was more conservative than IVW, thus the threshold for the p-value of the WM was still set at 0.05.

### 2.6 Sensitivity analysis

The interpretation of MR analysis results may be influenced by heterogeneity, horizontal pleiotropy, and correlated pleiotropy. Cochran’s Q test was employed to detect IV heterogeneity, and in case heterogeneity was observed (p<0.05), only multiplicative random-effects IVW could be used in MR analysis. Funnel plots were produced to represent the heterogeneity of IVs. To investigate horizontal pleiotropy (p<0.05), we conducted the MR-Egger intercept test. Despite our attempts to manage heterogeneity and correlated pleiotropy during the instrumental variable selection phase, we could not fully eliminate bias. Hence, we used a leave-one-out sensitivity analysis to ensure the stability of the MR analysis findings and prevent a single SNP from influencing the conclusions of the MR analysis. The results of the re-analysis of IVW were consistently significant each time an arbitrary SNP was removed, thus, demonstrating the robustness of the MR results. If the results were not significant after removing some SNPs, no dependable causal inference could be made.

## 3 Statistics and visualization

Given that we performed 68 two-sample MR analyses for each set of instrumental variables in the regional-level test, the threshold of p-value was corrected using the Bonferroni method and was set as 0.05/68 (p_adj_=7.35×10^-4^) for significant results. For the global-level test, considering both SA and TH directions, a significant p-value will be set as 0.05/2 ((p_adj_=0.025). A p-value of less than 0.05 was considered to be a nominally significant result. All statistical analyses were completed in R (version 4.2.1). Data visualization was accomplished with Adobe Illustrator CC 2019. The information for R packages could be checked in **Supplementary Materials**. Some of the image materials were from https://smart.servier.com.

## 4 Results

**Figure 1:**
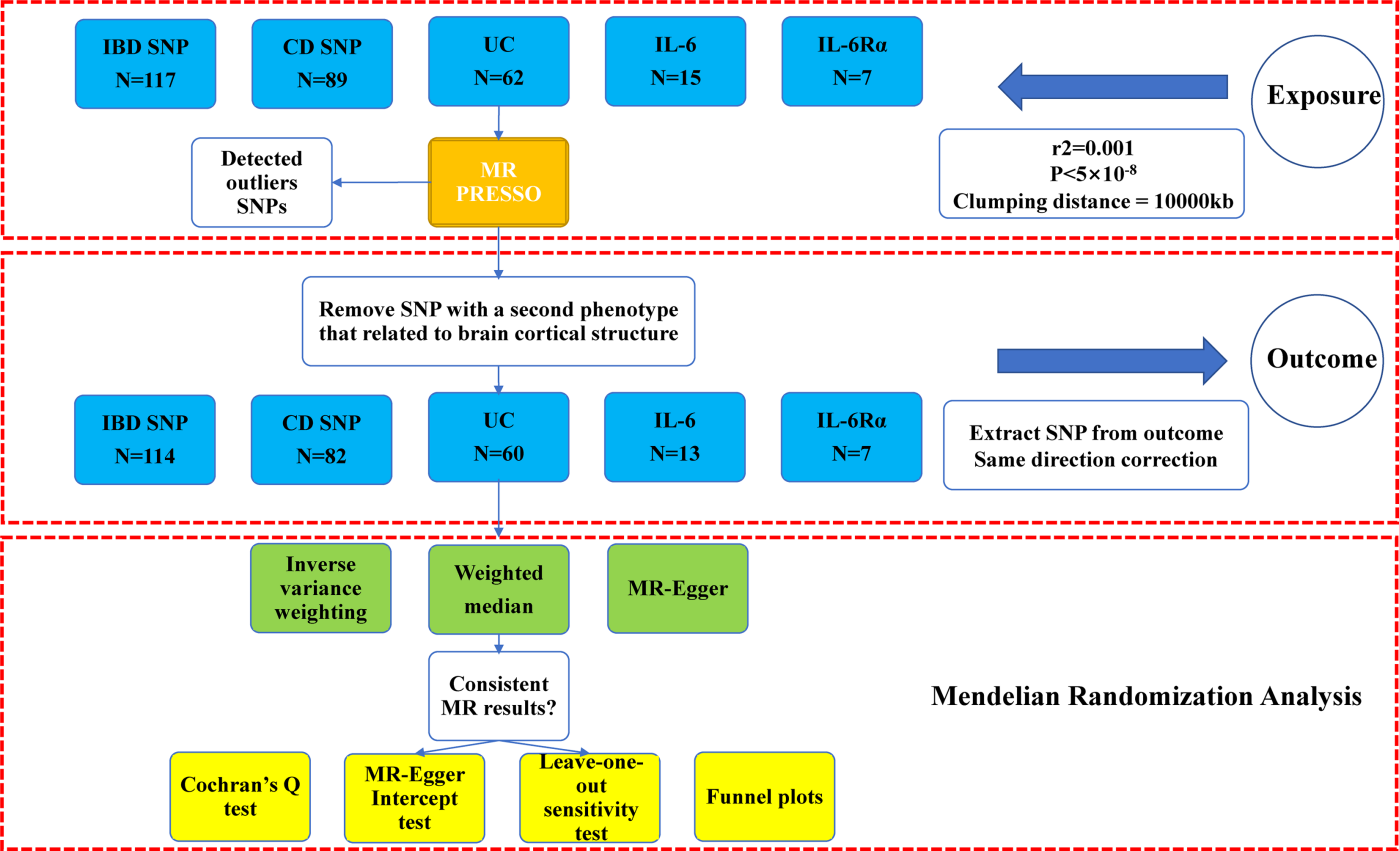
Study flow chart of the Mendelian randomization study revealing the causality between inflammatory bowel diseases, IL-6, and IL-6Rα and the brain cortical structure. IBD, inflammatory bowel disease; CD, Crohn’s disease; UC, Ulcerative colitis; IL-6, interleukin 6; IL-6Rα, interleukin-6 receptor subunit alpha. Outcome: brain cortical structure measured by magnetic resonance imaging were with global weighted.

The study process of this research was illustrated in **Figure 1**. Five sets of IVs were rigorously screened to represent IBD, CD, UC, IL-6 and IL-6Rα. Information on outliers excluded by MR PRESSO was in **Supplementary Materials**. In excluding the second phenotype, we removed rs6062496, rs4276914, rs4276914 from the IVs for IBD, rs6062496, rs492602, rs13107325, rs1583792, rs9258357, rs6808936, rs9482770 from CD, and rs9271176, rs6062496 from UC. We calculated the F-statistic value for every instrumental variable and every set of IVs, and they were all strong IVs (F>10). After ascertaining that all IVs were valid under existing conditions, we performed a two-sample Mendelian randomization analysis for the global and its 34 functional brain gyrus with inflammatory bowel diseases and inflammatory cytokines in two directions (SA and TH).

At the global level, neither inflammatory bowel diseases nor inflammatory cytokines (IL-6 and IL-6Rα) have been found to be causally associated with brain cortex structures (**Table 1**). There was a nominally significant association (β=526.336mm^2^, se=180.917mm^2^, p_ivw_=0.004) between IL-6Rα and global SA, but sensitivity analysis was insufficient to support a robust causal inference (**Supplementary Materials**).

**Table 1:** Significant and nominal significant results from Mendelian Randomization analysis.

	Exposure	Outcome	β_0_ (mm/mm^2^)	Se (mm/mm^2^)	IVW-derived p value	Cochran’s Q test derived p value	MR-Egger Intercept test derived p value	Leave-one-out analysis
Significant^**^	Crohn’s Disease	TH of pars orbitalis	-0.003	0.001	4.85×10^-04^	0.52	0.69	Yes
	IL-6	SA of middle temporal	-28.575	6.482	1.04×10^-05^	0.66	0.36	Yes
		TH of fusiform	0.008	0.002	8.86×10^-05^	0.92	0.52	Yes
		TH of pars opercularis	0.009	0.002	2.34×10^-04^	0.63	0.84	Yes
	IL-6Rα	SA of superior frontal	21.132	5.806	2.73×10^-04^	0.5	0.28	Yes
		TH of supramarginal	0.003	0.0002	7.86×10^-37^	1.00	0.88	Yes
Nominal Significant^*^	Inflammatory Bowel Disease	SA of inferior parietal	-8.162	2.96	0.006	0.82	0.9	Yes
		SA of pars triangularis	3.145	1.316	0.017	0.04	0.6	Yes
		TH of cuneus	0.002	0.001	0.041	0.16	0.47	No
		TH of paracentral	0.002	0.001	0.034	0.15	0.05	No
		TH of pars orbitalis	-0.003	0.001	0.011	0.7	0.89	Yes
		TH of postcentral	0.002	0.001	0.008	0.09	0.12	Yes
		TH of precentral	0.002	0.001	0.029	0.42	0.36	No
	Crohn’s Disease	TH of medial orbitofrontal	-0.002	0.001	0.034	0.15	0.95	No
		TH of paracentral	0.002	0.001	0.02	0.54	0.57	Yes
	Ulcerative Colitis	SA of bankssts	-1.635	0.81	0.043	0.95	0.33	No
		SA of frontal pole	0.678	0.248	0.006	0.67	0.85	Yes
		SA of fusiform	-3.91	1.879	0.037	0.91	0.22	No
		SA of insula	4.164	1.426	0.003	0.65	0.52	Yes
		TH of cuneus	0.002	0.001	0.037	0.98	0.60	No
		TH of fusiform	-0.002	0.001	0.04	0.66	0.56	No
		TH of isthmus cingulate	-0.004	0.002	0.037	0.10	0.44	No
		TH of postcentral	0.002	0.001	0.015	0.65	0.98	Yes
	IL-6	SA of temporal pole	2.635	0.948	0.005	0.92	0.42	Yes
		TH of entorhinal	0.024	0.011	0.032	0.19	0.77	No
		TH of pericalcarine	-0.007	0.003	0.01	0.67	0.40	No
		TH of superior parietal	-0.004	0.002	0.048	0.62	0.97	No
	IL-6Rα	Global SA	-526.336	180.917	0.004	0.67	0.38	No
		SA of fusiform	8.651	3.366	0.01	0.59	0.70	No
		SA of inferior parietal	-11.024	4.829	0.022	0.77	0.96	No
		SA of pars triangularis	2.351	1.039	0.024	0.98	0.64	No
		SA of precuneus	7.251	2.821	0.01	0.87	0.37	No
		SA of superior temporal	3.618	1.386	0.009	0.99	0.52	No
		TH of bankssts	0.004	0.001	0.002	0.84	0.50	No

^**^Significant results were described as results from consistency judgments, pleiotropy tests, and leave-one-out analysis. Consistency judgment should meet all of the following judgment criteria: (1) IVW-derived p value< 7.35×10^-4^; (2) WM-derived p value< 0.05; (3) WM and MR Egger derived β were in the same direction as the IVW. ^*^Nominal results were defined as IVW-derived p value< 0.05. Cochran’s Q test derived P value and MR-Egger intercept test derived P value< 0.05 is significant. “Yes” referred to result that causality was not driven by a specific SNP after leave-one-out analysis. IL-6, interleukin 6; IL-6Rα, interleukin-6 receptor subunit alpha; SA, surface area; TH, thickness. The above brain cortical structure measurements were with global weighted.

**Figure 2:**
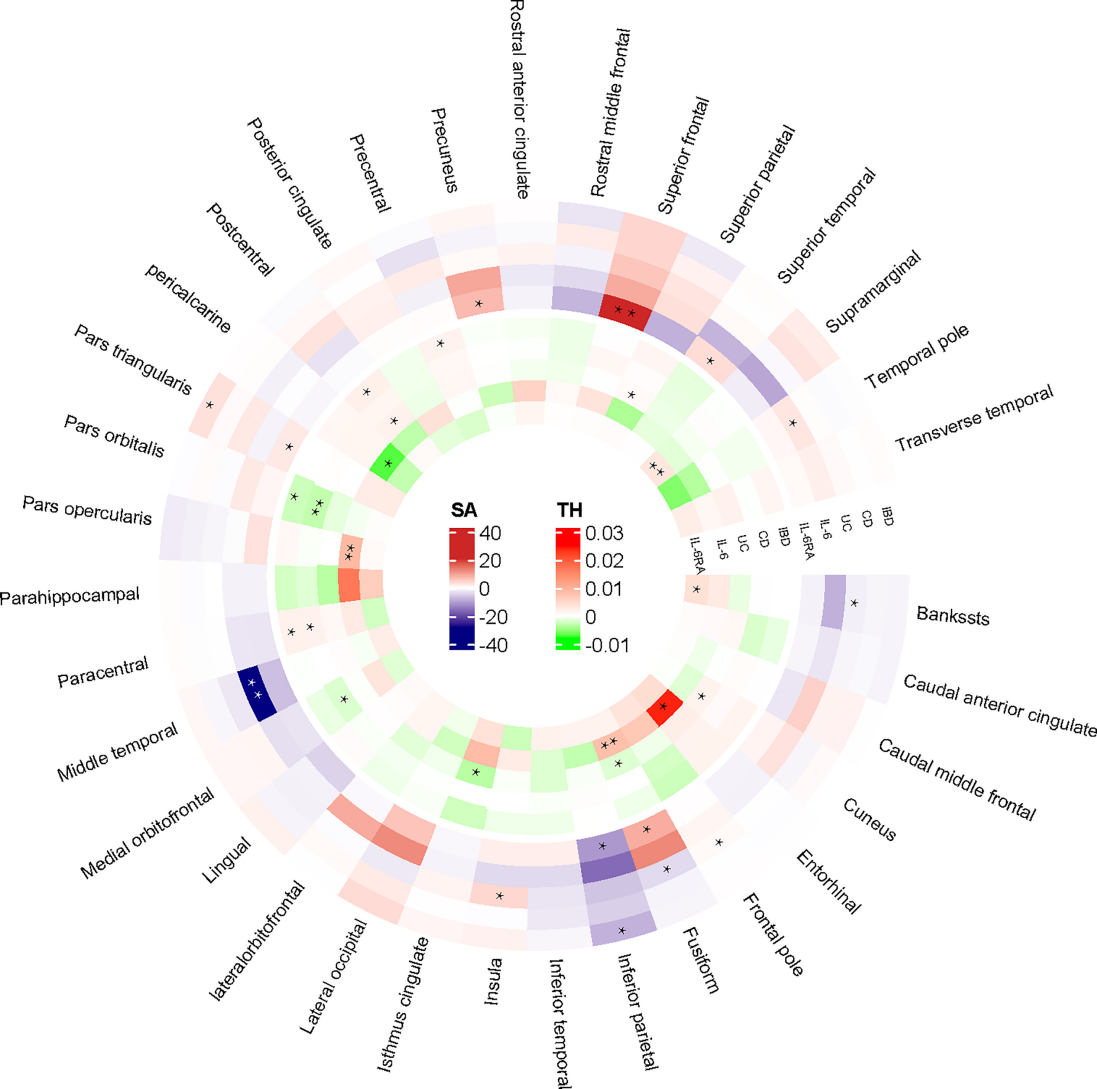
IVW-estimated effect values for altered cortical structures in 34 brain regions. “**” referred to the significant results from Mendelian Randomization analysis. “*” defined as the nominal significant results from Mendelian Randomization analysis. IBD, inflammatory bowel disease; CD, Crohn’s disease; UC, Ulcerative colitis; IL-6, interleukin 6; IL-6Rα, interleukin-6 receptor subunit alpha; SA, surface area; TH, thickness.

**Figure 3:**
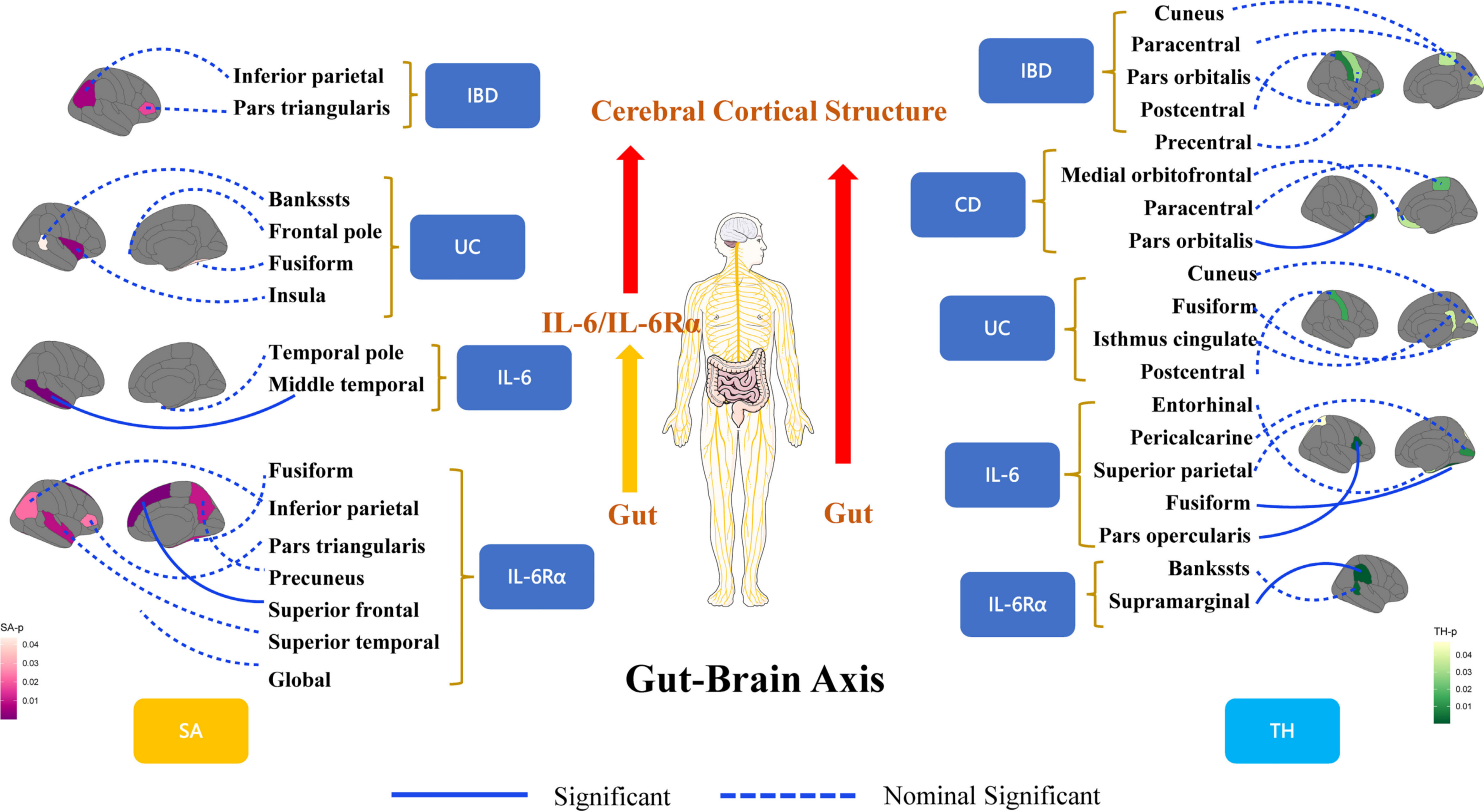
A framework for visualizing the results of Mendelian randomization analysis for revealing the connections of the gut-brain axis at the organic level. IBD, inflammatory bowel disease; CD, Crohn’s disease; UC, Ulcerative colitis; IL-6, interleukin 6; IL-6Rα, interleukin-6 receptor subunit alpha; SA, surface area; TH, thickness.

At the regional level, Crohn’s Disease was identified to decrease the TH of pars Orbitalis (β=-0.003mm, Se=0.001mm, p_ivw_ =4.85×10^-4^). The results of WM (p<0.05) and MR-Egger (consistent direction of β) supported causal interpretation and no heterogeneity or pleiotropy was detected. No outliers were detected from the leave-one-out sensitivity test and funnel plots (**Supplementary Materials**). There were potential causal links between IBD (CD and UC) and other functional brain gyri, but the current level of evidence was inadequate (**Figure 2**). Locations associated with inflammatory bowel diseases included bankssts, cuneus, fusiform, frontal pole, inferior parietal, insula, isthmus cingulate, medial orbitofrontal, paracentral, pars triangularis, pars orbitalis, postcentral, precentral. IL-6/IL-6Rα were more closely associated with the cerebral cortex (**Figure 3**). Increased IL-6 levels significantly diminished the SA of middle temporal (β=-28.575mm^2^, Se=6.482mm^2^, p_ivw_=1.04×10^-5^) but considerably increased the TH of fusiform (β=0.008mm, Se=0.002mm, p_ivw_=8.86×10^-5^) and pars opercularis (β=0.009mm, Se=0.002mm, p_ivw_=2.34×10^-4^). In parallel, an increase in IL-6Rα levels significantly increased the SA of superior frontal (β=21.132mm^2^, Se=5.806mm^2^, p_ivw_=2.73×10^-4^) and the TH of supramarginal (β=0.003mm, Se=0.0002mm, p_ivw_=7.86×10^-37^). All the above results were passed consistency analysis by WM and MR-Egger as well as sensitivity analysis, with heterogeneity and pleiotropy excluded (**Supplementary Materials**). Several suggestive sites were associated with IL-6/IL-6Rα, including bankssts, entorhinal, fusiform, inferior parietal, pars triangularis, pericalcarine, precuneus, superior parietal, superior temporal, and temporal pole (**Table 1**).

## 5 Discussion

Our study was the first using MR analysis to comprehensively reveal the causal relationship between inflammatory bowel diseases and brain cortical structure. We confirmed that genetic liability to Crohn’s Disease and high IL-6/IL-6Rα levels were causally associated with alterations in TH or SA in some brain regions, which further corroborated the organoleptic connection of the gut-brain axis.

In previous observational studies, patients with IBDs experienced a higher prevalence of psychiatric disorders (35, 36). A national cohort study from Sweden found that patients with IBD were at higher risk of developing multiple psychiatric disorders, such as mood disorders, major depressive disorder, and anxiety (37). A systematic review and meta-analysis also revealed that the prevalence of psychiatric comorbidities was high (11-82%) in patients with IBD (38). In addition to functional changes, organism changes in the brain have been observed in patients with IBDs. Wang et al. found that UC participants showed enhanced cortical stability in the medial prefrontal cortex (mPFC), which correlated with severe depression and anxiety-related measures (39). Bernstein et al. cortically mapped visceral pain in patients with gastrointestinal disorders by functional MRI and identified specific areas of activation (40). All of the above studies suggested a gut-brain interaction, but limitations such as inadequate sample size, difficulty in controlling for confounding factors, and unclear temporal sequence of causal events resulted in an insufficient level of evidence. Compared to traditional observational studies, MR studies could overcome confounding bias and thus make causal inferences (41). Only if the three major presuppositions of MR analysis were satisfied could the final causal inference be made (42). The selection of IVs influenced the reliability of causality to a large extent in MR analysis. Hence, we first calculated the F statistics of each SNP and every set of IVs to ensure they were highly correlated with the exposure factors. In our study, an F-statistic greater than 10 was considered a strong instrumental variable (43). In addition, we checked the second phenotype of every SNP in the PhenoScanner to exclude a potential alternative causal pathway that might lead to bias in causal inference. Finally, we verified the final causality by employing the consistency analysis and the sensitivity analysis.

The association of Crohn’s disease with cortical TH in pars orbitalis remained significant after correction of p-values (p<7.35×10^-4^) using the Bonferroni method. The pars orbitalis, along with the pars triangularis and pars opercularis, forms the frontal pole, which refers to the rostral portion of the inferior frontal gyrus in the frontal lobe of the brain. Belky et al. indicated that the pars orbitalis, located in the inferior frontal gyrus, was associated with semantics, emotion perception and emotional expression (44). In previous observational studies, brain regions of alteration in patients with IBD were concentrated in the frontal (superior and inferior) and middle temporal lobes (45–47). Li et al. reported functional alterations in the inferior frontal orbital cortex in patients with Crohn’s disease. Meanwhile, CD patients show abnormal neural activity in various areas of the brain associated with mood, pain and cognitive-related functions (48). Lener et al. found structural alterations in the ventral lateral prefrontal cortex of depressed patients, but limited to a reduction in cortical volume and surface area (49). However, most of these studies report changes in gray matter volume, while data on cortical thickness and cortical surface area are scarce and heterogeneous. Moreover, the sample sizes of all these cross-sectional studies were inadequate. Based on the substantial sample size and minimal confounding bias, MR studies can provide high levels of evidence for causal inference. Nevertheless, the underlying mechanisms leading to alterations in TH of pars orbitals in patients with Crohn’s disease still warrants further investigation.

In our study, high levels of IL-6/IL-6Rα were more closely associated with alterations in the regional structure of the brain, as demonstrated by the wider areas they affected. We used data from different sources and observed an association between IL-6 and cerebral cortical structures, which was similar to the findings of William et al (12). Our study simultaneously provides new evidence that inflammation was associated with alterations in cerebral cortical SA and TH. Additionally, the regions of the brain where high levels of IL-6 and IL-6Rα lead to structural changes in the cerebral cortex were also mainly located in the frontal and temporal lobes, which were involved in cognition, semantic processing and emotion (50). However, high IL-6 or IL-6Rα levels were not found to cause changes in the pars orbitalis, which implied that high IL-6 and IL-6Rα levels were not mediators of the changes in cerebral cortical structure caused by Crohn’s disease.

Inflammation was the primary pathological feature of IBDs. The levels of inflammatory cytokines were higher in patients with IBDs than in the normal population (51, 52). Inflammatory cytokines can be synthesized locally in the brain, and peripheral pro-inflammatory cytokines can also reach the brain *via* peripheral circulation (53). In particular, IL-6 had a broad range of biological effects and played an important role in the development of IBD (54). In our study, although high levels of the pro-inflammatory agent IL-6 and its receptors were not mediators of IBDs and structural alterations in the cerebral cortex, they themselves were causally associated with structural alterations in cortical areas. Wei et al. reported that overexpression of IL-6 resulted in impaired granule cell adhesion and migration, and a significant increase in IL-6 was detected in the cerebellum of autistic subjects (55). Previous studies have shown that inflammatory signals from the gut can also invoke apoptosis of astrocytes and oligodendrocytes and activate microglia, macrophages and endothelial cells in the brain, altering the excitability of the central nervous system (56). To some extent, these experimental studies illustrate the mechanisms of inflammation-induced structural changes in the cerebral cortex, but the specific mechanisms need to be comprehensively investigated. In addition to organism changes, functional changes due to inflammation have also been observed. An observational population-based study from Wiener et al. showed that serum IL-6 levels were significantly elevated in patients with mood disorders (57). MR study from Kelly et al. confirmed that high IL-6 signaling increased the risk of developing depression (58). Hence, another hypothesis suggested that the higher incidence of neuropsychiatric disorders in patients with IBD is partly or totally attributable to the repeated stimulation by high levels of inflammatory cytokines.

Other nominally significant results (IVW-derived p<0.05) should also be treated with caution. These nominally significant areas were also concentrated in brain regions associated with sensory, emotional, and semantic processing, such as the frontal lobe, temporal lobe, and pars triangularis (44). Moreover, some regions are involved in autonomic nervous system functions, such as the insula cortex, a major visceral sensory area that receives visceral tissue signal inputs from taste pathways, gastric mechanoreceptors, arterial chemoreceptors, and pressure receptors, and initiates and regulates various autonomic responses together with the medial prefrontal cortex (59). Structural abnormalities in these regions may be a precursor to functional abnormalities. Structural abnormalities in the above regions may lead to homeostatic dysregulation of the hypothalamic-pituitary-adrenal (HPA) axis and autonomic nervous system homeostasis. In addition, previous studies revealed that the presence of an imbalance between the HPA axis and autonomic tone in patients with IBD may contribute to their neuropsychiatric disorders (60, 61). This may partially explain the higher incidence of co-morbid neuropsychiatric disorders in patients with IBD.

Our study broadened the causal connection of the gut-brain axis at the organism level and reaffirmed that the lesions of inflammatory bowel disease are not limited to the gastrointestinal tract, especially Crohn’s disease, which is a systemic disease. Several limitations have to be listed. First, the population included in this study was almost derived from European cohorts, which limited the applicability of our results. Second, since we used publicly available summary data from the GWAS, the populations of exposures and outcomes may overlap. Therefore, we calculated the maximum overlap rate for this study from UK10K with UK biobank, which was 7.6%. Nevertheless, for our sample size, we consider this acceptable. Third, our study can only suggest a causal association between Crohn’s disease, IL-6 and IL-6R and alterations in cerebral cortical structures. However, whether the organism alterations will cause functional alterations or the threshold of functional alterations caused by organism alterations still needs to be further investigated. Image sectioning analysis of brain tissues would reveal promising findings which empower the concluded findings and more potential and more potential associations of inflammatory agents with cerebral cortical structures should be systematically explored.

## 6 Conclusion

A two-sample MR analysis comprehensively revealed that CD, IL-6 and IL-6Rα are causally responsible for alterations in regional cerebral cortical structure. Genetic liability for CD can causally result in reduced TH of pars orbitals. IL-6 and IL-6R would causally result in alterations in cerebral cortical structures in areas different from CD. Clinical patients with inflammatory bowel disease should focus on the long-term chronic management of inflammation, as organism changes may cause functional pathology. MRI may be added to the regular screening options for CD.

## Data availability statement

The datasets presented in this study can be found in online repositories. The names of the repository/repositories and accession number(s) can be found in the article/**Supplementary Material**.

## Ethics statement

This study used publicly available data from participant studies on human subjects approved by the Ethical Standards Committee.

## Author contributions

CL and SZ conceived the study design and wrote the first manuscript for the study. JY, KL, and JZ verified the underlying data. CL, KR, and SZ were responsible for data acquisition and analysis. SZ, CL, and JZ conducted the statistical analysis. SZ, CL, JZ, KR, KL, and JY participated in data interpretation and visualization. CL, SZ, JZ and JY do the major revision of the manuscript for content. All authors contributed to the article and approved the submitted version.
