# Supplementary material for: Inflammatory bowel diseases, interleukin-6 and interleukin-6 receptor subunit alpha in causal association with cerebral cortical structure: a Mendelian randomization analysis
Source: Front Immunol. 2023 Apr 20;14:1154746. doi: 10.3389/fimmu.2023.1154746 (PMC10157470; doi:10.3389/fimmu.2023.1154746)
Supplement: Supplementary file 1 [file DataSheet_1.pdf]

Supplementary materials:

(1) Data visualization information

(2) Sensitivity analysis

(3) Heterogeneity analysis.

Inflammatory bowel diseases, interleukin-6 and interleukin-6 receptor subunit alpha in causal association with cerebral cortical structure: A Mendelian Randomization Analysis

Chunlong Liu [a 1], Shijie Zhu [b 1], Jian Zhang [c 1], Kuiwu Ren [a], Kangkang Li [d], Jiangtao Yu [a *]

Abbreviations

MR: Mendelian Randomization

IBD: inflammatory bowel disease

CD: Crohn’s disease

UC: Ulcerative colitis

SA: surface area

TH: thickness

IL-6: interleukin 6

IL-6α: interleukin 6 receptor subunit α

IIBDGC: International Inflammatory Bowel Disease Genetics Consortium

ENIGMA: The Enhancing Neuroimaging Genetics through Meta-Analysis Consortium

MR PRESSO: Mendelian randomization pleiotropy residual sum and outlier

Table S1. Data Visualization tools.

Table S2. Descriptions of study cohorts.

Table S3. 114 index SNPs represented genetically predicted IBD.

Table S4. 82 index SNPs represented genetically predicted CD.

Table S5. 60 index SNPs represented genetically predicted UC.

Table S6. 13 index SNPs represented genetically predicted IL-6 levels.

Table S7. 7 index SNPs represented genetically predicted IL-6α.

Table S8. Information of outliers detected by MR PRESSO.

Figure S1. Scatter plots of significant results from MR analysis.

Figure S2. Leave-one-out analysis of significant results from MR analysis.

Figure S3. Funnel plots of significant results from MR analysis.

Figure S4. Scatter plots of nominal significant results from genetically predicted IBD on brain cortical structures.

Figure S5. Leave-one-out analysis of nominal significant results from genetically predicted IBD on brain cortical structures.

Figure S6. Funnel plots of nominal significant results from genetically predicted IBD on brain cortical structures.

Figure S7. Sensitivity analysis of nominal significant results from genetically predicted CD on brain cortical structures.

Figure S8. Scatter plots of nominal significant results from genetically predicted UC on brain cortical structures.

Figure S9. Leave-one-out analysis of nominal significant results from genetically predicted UC on brain cortical structures.

Figure S10. Funnel plots of nominal significant results from genetically predicted UC on brain cortical structures.

Figure S11. Sensitivity analysis of nominal significant results from genetically predicted IL-6 on brain cortical structures.

Figure S12. Sensitivity analysis of nominal significant results from genetically predicted IL-6 on brain cortical structures.

Figure S13. Scatter plots of nominal significant results from genetically predicted IL-6α on brain cortical structures.

Figure S14. Leave-one-out analysis of nominal significant results from genetically predicted IL-6α on brain cortical structures.

Figure S15. Funnel plots of nominal significant results from genetically predicted IL-6α on brain cortical structures.

Table S1. Data Visualization tools.

Software and algorithms	SOURCE	IDENTIFIER
R 4.1.2	R Foundation for Statistical Computing	https://www.r-project.org/
Adobe illustrator CC 2019	Adobe company	https://www.adobe.com/cn
devtools 2.4.4	Jennifer Bryan et.al	https://CRAN.R-project.org/package=devtools
remotes 2.4.2	Gábor Csárdi et.al	https://CRAN.R-project.org/package=remotes
TwoSampleMR 0.5.6	Gibran Hemani	https://mrcieu.github.io/TwoSampleMR/articles/introduction.html
plyr 1.8.7	Hadley Wickham	https://CRAN.R-project.org/package=plyr
circlize 0.4.15	Zuguang Gu	https://CRAN.R-project.org/package=circlize
ComplexHeatmap 2.13.1	Zuguang Gu	https://github.com/jokergoo/ComplexHeatmap
ggplot2 3.3.6	Hadley Wickham et.al	https://CRAN.R-project.org/package=ggplot2
ggseg 1.6.5	Athanasia Mo Mowinckel et.al	https://CRAN.R-project.org/package=ggseg

Table S2. Descriptions of study cohorts.

Major Study name	PMID	Category	Total	Major Ancestry	Detailed	Cohort/study names	PMID of cohort/study	N	case	control	Ancestry
Genome-wide association study implicates immune activation of multiple integrin genes in inflammatory bowel disease	28067908	IBD	59957	European	New added data	UK low coverage whole genome sequencing IBD study;UK HumanCoreExome genotyped IBD study	28067908	25305	12160	13145	European
				European	Old data	IBDGC	26192919	34652	12882	21770	European
		CD	40226	European	New added data	UK low coverage whole genome sequencing IBD study;UK HumanCoreExome genotyped IBD study		19343	6238	13105	European
				European	Old data	IBDGC		20883	5956	14927	European
		UC	45975	European	New added data	UK low coverage whole genome sequencing IBD study;UK HumanCoreExome genotyped IBD study		18543	5398	13145	European
				European	Old data	IBDGC		27432	6968	20464	European
The genetic architecture of the human cerebral cortex	32193296		51665	European	principal	1000BRAINS		775	775	NA	European
						ADNI1		735	735	NA	European
						ADNI2GO		649	649	NA	European
						ALSPACa		391	391	NA	European

	ASRB	233	233	NA	European
	BETULA	311	311	NA	European
	BIG-Affy	1180	1180	NA	European
	BIG-PsychChip	432	432	NA	European
	BONN	102	102	NA	European
	BrainScale	242	242	NA	European
	CARDIFF	270	270	NA	European
	DNS-V3	324	324	NA	European
	DNS-V4	191	191	NA	European
	EPIGEN	178	178	NA	European
	FOR2107	785	785	NA	European
	GIG	283	283	NA	European
	GSP	442	442	NA	European
	HUBIN	177	177	NA	European
	HUNT	876	876	NA	European
	IMAGEN	1358	1358	NA	European
	IMpACT	238	238	NA	European
	LBC1936	604	604	NA	European
	LIBD	484	484	NA	European
	MCIC	162	162	NA	European
	MooDS	282	282	NA	European
	MPIP	550	550	NA	European
	MPRC	387	387	NA	European
	MÜNSTER	985	985	NA	European
	NCNG	321	321	NA	European

	NESDA	254	254	NA	European
	NeuroIMAGE	210	210	NA	European
	NTR	322	322	NA	European
	OATS	360	360	NA	European
	PAFIP	112	112	NA	European
	PDNZ	164	164	NA	European
	PING	337	337	NA	European
	PPMI	414	414	NA	European
	QTIM	996	996	NA	European
	SHIP	1118	1118	NA	European
	SHIP-Trend	891	891	NA	European
	Sydney MAS	494	494	NA	European
	SYS	1675	1675	NA	European
	TCD-NUIG	192	192	NA	European
	TOP	505	505	NA	European
	TOP3T	400	400	NA	European
	UiO2016	229	229	NA	European
	UiO2017	308	308	NA	European
	UMCU	698	698	NA	European
	FBIRN	283	283	NA	Mixed
	GOBS	1272	1272	NA	Mexican American
	IMH	152	152	NA	Singapore
	Meth-CT	130	130	NA	South African

					MIRECC		253	253	NA	European and African American
					OSAKA		401	401	NA	Japanese
					UNICAMP		141	141	NA	Brazil
					PING-nonEUR		331	331	NA	Non European
					UKBB-nonEUR		264	264	NA	Non European
					UKBB		10083	10083	NA	European
				Repulication	ID1000		777	777	NA	European
					CHARGE		13952	13952	NA	Mixed
Genomic and drug target evaluation of 90 cardiovascular proteins in 30,931 individuals	33067605	21758	European	principal	IMPROVE	23152477	3700	3430	NA	European
					STANLEY		10000	681	NA	Swedish
					EpiHealth	23435790	2500	2335	NA	European
					PIVUS	16141402	1016	933	NA	European
					ULSAM	16030278	1221	730	NA	European
					INTERVAL	27863252	4994	4,987	NA	European
					LifeLines-DEEP	26319774	1539	1178	NA	European
					NSPHS	19060911 & 20568910	1067	965	NA	European

	STABILITY	24678955	15828	2,967	NA	Worldwide (78% reported with ethnic group white in main trial)
	Estonian BB	24518929	500	496	NA	European
	ORCADES	18760389	2078	971	NA	European
	VIS		960		NA	European
	MPP-RES	20211303	1792	882	NA	European

Table S3. 114 index SNPs represented genetically predicted IBD.

se.exposure	chr.exposure	pval.exposure	beta.exposure	samplesize.exposure	pos.exposure	SNP	effect_allele.exposure	other_allele.exposure	eaf.exposure	R2	F-statistic
0.0142	1	1.02E-09	0.087	59957	1.73E+08	rs12136659	C	T	NA	0.000626	37.53594
0.0149	1	3.63E-11	0.0985	59957	1.98E+08	rs2488398	C	G	NA	0.000728	43.7004
0.0164	1	1.58E-15	0.1308	59957	8168261	rs10746475	A	T	NA	0.00106	63.60823
0.0124	1	4.8E-21	-0.1172	59957	20227723	rs4654925	C	G	NA	0.001488	89.33001
0.0332	1	2.89E-08	-0.1844	59957	39802381	rs112936798	C	A	NA	0.000514	30.8483
0.014	1	7.5E-22	-0.1346	59957	2.01E+08	rs35730213	C	G	NA	0.001539	92.43141
0.0165	1	4.04E-31	0.1911	59957	2.07E+08	rs3024493	A	C	NA	0.002232	134.134
0.0124	1	4.46E-10	0.0773	59957	67656286	rs11209013	G	A	NA	0.000648	38.85985
0.0294	1	4.6E-111	-0.6578	59957	67707690	rs11581607	A	G	NA	0.00828	500.5859
0.0128	1	2.98E-11	-0.0848	59957	1.51E+08	rs1336900	A	G	NA	0.000731	43.88916
0.0133	1	1.94E-20	-0.123	59957	1.61E+08	rs10800309	G	A	NA	0.001424	85.52488
0.0163	1	2.75E-08	0.0907	59957	1215424	rs1268339	C	T	NA	0.000516	30.96171
0.016	1	3.79E-13	0.1164	59957	20140036	rs1317209	A	G	NA	0.000882	52.92386
0.014	1	1.72E-10	-0.0892	59957	20142413	rs3820330	A	C	NA	0.000677	40.59375
0.0134	1	3.83E-09	0.0789	59957	1.61E+08	rs7532133	G	A	NA	0.000578	34.66809
0.0126	2	1.37E-13	-0.0931	59957	28614401	rs11677002	C	T	NA	0.00091	54.59386
0.018	2	5.45E-13	0.1298	59957	43851246	rs55946629	A	C	NA	0.000867	51.99839
0.013	2	7.63E-15	0.1011	59957	2.42E+08	rs4676408	A	G	NA	0.001008	60.47852
0.0126	2	1.67E-28	0.1395	59957	61204641	rs7608697	C	A	NA	0.00204	122.5724
0.0205	2	2.56E-08	-0.1143	59957	1.88E+08	rs13422838	C	T	NA	0.000518	31.08639
0.0132	2	1.55E-09	-0.0797	59957	2E+08	rs62180107	C	G	NA	0.000608	36.45476

0.0124	2	5.12E-29	0.1391	59957	2.34E+08	rs3792111	T	C	NA	0.002094	125.8335
0.0123	2	8.9E-12	-0.0843	59957	1.03E+08	rs1558619	T	G	NA	0.000783	46.97107
0.0151	2	4.65E-11	0.0996	59957	25195577	rs76286777	C	T	NA	0.000725	43.50611
0.0202	2	2.3E-08	-0.1129	59957	1.45E+08	rs72852162	C	A	NA	0.000521	31.2371
0.0125	2	1.22E-13	-0.0924	59957	1.82E+08	rs6740847	G	A	NA	0.000911	54.63984
0.0125	2	4.49E-10	-0.078	59957	2.19E+08	rs62183956	T	C	NA	0.000649	38.9363
0.0131	3	1.22E-35	0.1635	59957	49714225	rs1131095	C	T	NA	0.002591	155.7681
0.0163	3	9.27E-10	-0.1	59957	1.88E+08	rs56116661	T	C	NA	0.000627	37.63659
0.0417	3	3.72E-08	0.2293	59957	17184935	rs77272631	C	G	NA	0.000504	30.2358
0.0124	3	2.67E-08	-0.0692	59957	1.01E+08	rs503734	G	A	NA	0.000519	31.14256
0.014	3	2.54E-09	-0.0832	59957	71175495	rs2593855	T	C	NA	0.000589	35.31637
0.0127	4	4.8E-08	0.0694	59957	38588453	rs11734570	A	G	NA	0.000498	29.8605
0.0127	4	2.67E-12	0.0886	59957	1.24E+08	rs62324212	A	C	NA	0.000811	48.66823
0.0155	5	3.37E-14	-0.1179	59957	40207142	rs4957256	T	C	NA	0.000964	57.85618
0.0125	5	5.17E-09	0.0731	59957	1.5E+08	rs17656349	T	C	NA	0.00057	34.19796
0.0189	5	4.01E-11	0.125	59957	1.5E+08	rs6579807	T	C	NA	0.000729	43.74032
0.0127	5	3.48E-40	0.1689	59957	40414419	rs1445004	T	C	NA	0.002941	176.8632
0.0142	5	4.23E-08	-0.0776	59957	1.59E+08	rs62378712	C	T	NA	0.000498	29.86292
0.0128	5	6.15E-13	-0.0919	59957	96247810	rs6873866	C	T	NA	0.000859	51.54619
0.0129	5	1.95E-10	0.0819	59957	1.42E+08	rs10041497	C	T	NA	0.000672	40.30639
0.0134	5	1.59E-39	0.1767	59957	1.59E+08	rs755374	T	C	NA	0.002892	173.8798
0.0138	5	1.77E-10	0.0877	59957	1.77E+08	rs56235845	G	T	NA	0.000673	40.3856
0.0125	5	1.1E-27	0.1366	59957	1.32E+08	rs11739135	C	G	NA	0.001988	119.4172
0.0124	5	1.45E-08	0.0702	59957	1.12E+08	rs341295	T	C	NA	0.000534	32.04914
0.0133	6	1.56E-14	0.1019	59957	1.06E+08	rs11152949	G	A	NA	0.000978	58.69899

0.0159	6	3.39E-11	0.1053	59957	14716017	rs1267496	C	G	NA	0.000731	43.85792
0.0476	6	4.73E-73	0.86	59957	32247045	rs145568234	G	T	NA	0.005415	326.4138
0.0153	6	3.75E-28	-0.1687	59957	32773497	rs6457681	T	G	NA	0.002024	121.5718
0.0152	6	7.14E-12	0.1043	59957	20678430	rs4712528	C	G	NA	0.000785	47.08331
0.036	6	3.14E-15	0.2836	59957	31492353	rs143210366	G	T	NA	0.001034	62.05731
0.0129	6	2.4E-10	-0.0818	59957	90931858	rs62408218	T	C	NA	0.00067	40.20802
0.013	6	1.06E-08	-0.0743	59957	1.59E+08	rs212402	A	G	NA	0.000545	32.66453
0.0237	6	2.28E-11	-0.1583	59957	31923654	rs34140409	T	C	NA	0.000744	44.61189
0.0149	6	6.64E-09	0.0863	59957	1.38E+08	rs6933404	C	T	NA	0.000559	33.54552
0.0123	6	1.16E-18	0.1088	59957	1.67E+08	rs35171809	G	A	NA	0.001303	78.24076
0.0127	7	4.94E-16	-0.1033	59957	1.07E+08	rs10953551	G	A	NA	0.001102	66.15744
0.0128	7	3.04E-10	-0.0805	59957	1.48E+08	rs243505	G	A	NA	0.000659	39.55099
0.0242	7	3.26E-08	-0.1338	59957	20577298	rs149169037	A	G	NA	0.00051	30.568
0.0133	7	4.5E-11	0.0879	59957	50304461	rs1456896	T	C	NA	0.000728	43.67772
0.0131	7	1.97E-08	-0.0737	59957	1.01E+08	rs62482552	A	G	NA	0.000528	31.6503
0.0152	7	3.88E-08	-0.0837	59957	6545188	rs11768365	G	A	NA	0.000505	30.3214
0.0669	8	8.95E-09	-0.3848	59957	21986850	rs78771661	T	C	NA	0.000551	33.08289
0.0127	8	1.12E-12	0.0907	59957	1.27E+08	rs4380956	A	G	NA	0.00085	51.00264
0.0189	8	1.41E-08	-0.1074	59957	1.3E+08	rs938650	A	G	NA	0.000538	32.29018
0.0131	9	2.46E-36	-0.1643	59957	4984530	rs1887428	C	G	NA	0.002617	157.2961
0.0137	9	4.1E-27	0.1475	59957	1.18E+08	rs10114470	C	T	NA	0.00193	115.912
0.0125	9	3.52E-36	0.1574	59957	1.39E+08	rs3829110	G	A	NA	0.002638	158.5532
0.0138	10	1.11E-12	-0.098	59957	81042475	rs1250573	A	G	NA	0.00084	50.4289
0.0136	10	3.99E-13	-0.099	59957	30690376	rs10826797	T	G	NA	0.000883	52.98807
0.0124	10	1.19E-34	-0.152	59957	1.01E+08	rs6584282	G	A	NA	0.0025	150.2551

0.0133	10	2.74E-09	0.0792	59957	1.12E+08	rs11195128	T	C	NA	0.000591	35.4595
0.0131	10	3.12E-13	0.0951	59957	35492832	rs2384352	G	A	NA	0.000878	52.69919
0.0126	10	2.3E-36	0.1585	59957	64445564	rs10761659	G	A	NA	0.002632	158.2351
0.0125	10	1.38E-08	0.071	59957	94429467	rs7918084	T	C	NA	0.000538	32.26132
0.017	10	1.18E-09	-0.1031	59957	1.26E+08	rs111456533	A	G	NA	0.000613	36.77943
0.0148	11	2.44E-08	0.0827	59957	1.28E+08	rs11221335	C	T	NA	0.000521	31.22289
0.0125	11	7.19E-33	0.1488	59957	76299649	rs11236797	A	C	NA	0.002358	141.7005
0.0411	12	4.53E-17	0.3452	59957	40822098	rs117981694	A	G	NA	0.001175	70.5413
0.0127	12	1.27E-25	0.1324	59957	68492980	rs12825700	A	G	NA	0.001809	108.6811
0.0145	13	1.9E-11	0.0971	59957	27542030	rs3897234	C	T	NA	0.000747	44.84231
0.0305	13	1.13E-09	-0.1857	59957	40836270	rs140933577	C	T	NA	0.000618	37.0689
0.0124	14	1.84E-11	0.0833	59957	69282887	rs194746	T	C	NA	0.000752	45.12655
0.0207	14	1.1E-13	0.1536	59957	88417517	rs3850378	C	T	NA	0.000917	55.05886
0.1782	15	6.27E-14	1.3366	59957	80205354	rs1864239	G	A	NA	0.000937	56.25654
0.0145	15	1.37E-21	0.1382	59957	67455630	rs56062135	T	C	NA	0.001513	90.83759
0.0155	16	2.06E-08	-0.0872	59957	23855853	rs7190426	C	A	NA	0.000528	31.64864
0.0137	16	6.55E-16	-0.1105	59957	28489342	rs28374519	A	G	NA	0.001084	65.05324
0.0172	16	8.77E-11	-0.1116	59957	50383077	rs9934775	T	C	NA	0.000702	42.09757
0.0327	16	2.99E-17	0.2765	59957	50885211	rs8056255	A	T	NA	0.001191	71.49576
0.0362	16	5.18E-11	-0.2374	59957	81916912	rs11548656	G	A	NA	0.000717	43.00607
0.0138	16	7.83E-46	0.1961	59957	50758849	rs749910	A	G	NA	0.003357	201.9215
0.0126	16	4.96E-10	0.0783	59957	11367477	rs2301127	A	G	NA	0.000644	38.61606
0.0169	16	2.5E-11	0.113	59957	86014241	rs16940202	C	T	NA	0.000745	44.70633
0.0124	17	7.73E-30	0.1406	59957	38043649	rs12936409	T	C	NA	0.00214	128.562
0.0126	17	1.34E-18	-0.1109	59957	40514201	rs744166	G	A	NA	0.00129	77.46535

0.0139	17	6.23E-12	-0.0959	59957	32617265	rs714910	C	A	NA	0.000793	47.59848
0.018	17	2.47E-13	-0.1319	59957	70611194	rs113846785	CG	C	NA	0.000895	53.69454
0.0147	18	7.5E-09	-0.0851	59957	46404813	rs1319951	G	C	NA	0.000559	33.51274
0.019	18	1.04E-16	0.1581	59957	12818922	rs80262450	A	G	NA	0.001153	69.23761
0.0152	19	4.24E-17	0.1281	59957	1123378	rs4807569	C	A	NA	0.001183	71.0226
0.0276	19	1.63E-09	-0.1665	59957	10626375	rs7256518	A	G	NA	0.000607	36.39115
0.0166	19	2.6E-17	0.1407	59957	33748183	rs62126610	G	A	NA	0.001197	71.83855
0.0157	19	1.84E-12	-0.1107	59957	10496621	rs11669299	T	C	NA	0.000829	49.71431
0.0138	20	1.23E-08	0.0786	59957	6093889	rs4256018	G	T	NA	0.000541	32.43937
0.0135	20	1.07E-17	0.1156	59957	43065028	rs6017342	C	A	NA	0.001221	73.32189
0.0134	20	4.55E-08	-0.0734	59957	48955595	rs6063502	G	A	NA	0.0005	30.00323
0.0132	20	7.38E-10	-0.0813	59957	57855032	rs154873	A	G	NA	0.000632	37.93314
0.0126	21	3.98E-31	-0.1462	59957	16816017	rs1297264	G	A	NA	0.00224	134.6292
0.0146	21	1.96E-29	-0.1643	59957	40466299	rs2836881	T	G	NA	0.002108	126.6353
0.0125	21	1.83E-24	-0.128	59957	45613825	rs2838517	C	T	NA	0.001746	104.8541
0.0171	22	4.6E-24	-0.1732	59957	39659773	rs2413583	T	C	NA	0.001708	102.5862
0.016	22	7.14E-16	0.1293	59957	21916166	rs5754100	C	T	NA	0.001088	65.30442
0.013	22	1.47E-08	0.0734	59957	30526632	rs5763793	T	G	NA	0.000531	31.87799

Table S4. 82 index SNPs represented genetically predicted CD.

se.exposure	samplesize.exposure	pval.exposure	beta.exposure	pos.exposure	chr.exposure	SNP	effect_allele.exposure	other_allele.exposure	eaf.exposure	R2	F-statistic
0.0174	40266	3.99E-10	-0.1088	1.55E+08	1	rs12131079	T	C	NA	0.00097	39.09649
0.0181	40266	1.17E-10	-0.1166	2.01E+08	1	rs35730213	C	G	NA	0.00103	41.49716
0.0227	40266	1.24E-14	-0.1748	2.07E+08	1	rs3122605	A	G	NA	0.00147	59.29384
0.0384	40266	5.11E-09	-0.2245	1.61E+08	1	rs114802258	T	C	NA	0.000848	34.17816
0.0189	40266	7.74E-12	-0.1292	1.98E+08	1	rs4316387	C	T	NA	0.001159	46.72829
0.0286	40266	1.77E-15	-0.2275	1.14E+08	1	rs6679677	A	C	NA	0.001569	63.27165
0.0181	40266	5.1E-22	0.1748	1.73E+08	1	rs6704109	T	C	NA	0.002311	93.26187
0.0165	40266	5.84E-97	-0.3447	67681669	1	rs7517847	G	T	NA	0.010722	436.4084
0.0185	40266	9.29E-14	-0.1376	1.03E+08	2	rs11378157	AG	A	NA	0.001372	55.31868
0.038	40266	1.75E-08	-0.2144	1.46E+08	2	rs11683692	C	T	NA	0.00079	31.83177
0.0162	40266	3.5E-12	0.1123	25161236	2	rs4343432	G	A	NA	0.001192	48.05161
0.0163	40266	4.57E-12	-0.1124	28614401	2	rs11677002	C	T	NA	0.00118	47.54839
0.0179	40266	2E-12	0.1258	2.31E+08	2	rs34004493	G	A	NA	0.001225	49.38939
0.0162	40266	1.51E-62	0.2704	2.34E+08	2	rs3816234	A	G	NA	0.006871	278.5876
0.0231	40266	2.85E-14	0.1755	43851246	2	rs55946629	A	C	NA	0.001431	57.71766
0.0163	40266	4.03E-14	0.1229	61204641	2	rs7608697	C	A	NA	0.00141	56.84693
0.0161	40266	9.72E-11	-0.104	1.82E+08	2	rs6740847	G	A	NA	0.001035	41.72471
0.0212	40266	5.67E-10	-0.1312	1.88E+08	3	rs56116661	T	C	NA	0.00095	38.29785
0.017	40266	3.77E-24	0.1722	49697459	3	rs9836291	A	G	NA	0.002542	102.5999
0.0162	40266	6.46E-09	-0.0941	53133149	3	rs2581828	G	C	NA	0.000837	33.73865
0.0212	40266	4.12E-08	0.1164	26047616	4	rs73243877	G	A	NA	0.000748	30.14482

0.0163	40266	8.02E-11	0.106	1.24E+08	4	rs62324212	A	C	NA	0.001049	42.28779
0.0244	40266	3.44E-16	0.1993	1.5E+08	5	rs6579807	T	C	NA	0.001654	66.71345
0.0174	40266	1.38E-29	0.1969	1.59E+08	5	rs755374	T	C	NA	0.00317	128.0476
0.0166	40266	8.26E-56	0.2605	40411291	5	rs6451494	C	T	NA	0.006079	246.2508
0.0243	40266	3.61E-11	-0.1612	40530242	5	rs112856973	C	T	NA	0.001092	44.00439
0.0164	40266	1.35E-15	-0.1314	96247810	5	rs6873866	C	T	NA	0.001592	64.19208
0.016	40266	5.59E-36	0.2004	1.32E+08	5	rs2188962	T	C	NA	0.003881	156.8678
0.0167	40266	3.24E-12	0.1162	1.42E+08	5	rs181826	A	C	NA	0.001201	48.41253
0.0198	40266	7.01E-11	0.1291	20674811	6	rs1012636	T	G	NA	0.001055	42.51092
0.0172	40266	1.18E-09	-0.1049	91011673	6	rs1321859	T	C	NA	0.000923	37.19397
0.0169	40266	4.04E-17	0.1423	1.06E+08	6	rs73516754	C	A	NA	0.001758	70.89487
0.0159	40266	9.07E-23	0.1566	1.67E+08	6	rs35171809	G	A	NA	0.002403	96.9991
0.0316	40266	4.17E-18	0.2745	31367874	6	rs111281598	C	T	NA	0.001871	75.45516
0.0273	40266	2.39E-09	0.163	32684630	6	rs6941902	C	T	NA	0.000885	35.64744
0.0163	40266	1.39E-09	-0.0989	21441035	6	rs7753014	G	C	NA	0.000913	36.81254
0.0633	40266	4.31E-42	0.8602	32247045	6	rs145568234	G	T	NA	0.004565	184.6587
0.0432	40266	2.57E-12	0.3027	32450319	6	rs9501641	T	C	NA	0.001218	49.09483
0.0162	40266	1.49E-11	-0.1096	1.59E+08	6	rs212409	A	G	NA	0.001135	45.7688
0.0173	40266	8.73E-12	0.1183	50306780	7	rs9656588	C	T	NA	0.00116	46.75798
0.0247	40266	1.65E-12	-0.1747	1.3E+08	8	rs938650	A	G	NA	0.001241	50.02307
0.0165	40266	1.15E-15	0.132	1.27E+08	8	rs4380956	A	G	NA	0.001587	63.99682
0.0344	40266	8.9E-11	0.2234	1.45E+08	8	rs79832570	C	T	NA	0.001046	42.17236
0.0177	40266	1.76E-21	0.1687	1.18E+08	9	rs10114470	C	T	NA	0.002251	90.83685
0.0169	40266	8.54E-23	-0.166	4984530	9	rs1887428	C	G	NA	0.00239	96.47642
0.0162	40266	3.14E-30	0.1848	1.39E+08	9	rs4077515	T	C	NA	0.003221	130.1225

0.0171	40266	4.13E-11	0.1131	1.12E+08	10	rs10884966	A	G	NA	0.001085	43.74329
0.0261	40266	1.98E-08	0.1468	6094697	10	rs61839660	T	C	NA	0.000785	31.63367
0.0189	40266	8.31E-12	-0.1293	30805480	10	rs2002695	G	A	NA	0.001161	46.80065
0.0162	40266	2.35E-29	0.1827	64438771	10	rs10822050	C	T	NA	0.003149	127.182
0.0161	40266	2.89E-11	0.1074	75655628	10	rs2675670	C	G	NA	0.001104	44.49746
0.0167	40266	2.09E-15	-0.1323	35496626	10	rs1148246	T	C	NA	0.001556	62.75743
0.0179	40266	1.92E-17	-0.1522	81042475	10	rs1250573	A	G	NA	0.001792	72.2939
0.016	40266	3.44E-25	-0.1658	1.01E+08	10	rs6584282	G	A	NA	0.00266	107.3761
0.0161	40266	8.51E-28	0.176	76299649	11	rs11236797	A	C	NA	0.002959	119.4956
0.0178	40266	1.06E-09	0.1083	6493100	12	rs28999107	T	G	NA	0.000919	37.0165
0.0185	40266	4.13E-09	-0.1089	1.13E+08	12	rs77566919	A	G	NA	0.00086	34.649
0.0504	40266	1.95E-21	0.4794	40824663	12	rs34635748	T	C	NA	0.002242	90.47184
0.0189	40266	9.11E-14	0.141	44475398	13	rs1373904	G	A	NA	0.00138	55.65357
0.0161	40266	1.24E-09	0.0975	69282887	14	rs194746	T	C	NA	0.00091	36.67211
0.0267	40266	8.31E-14	0.199	88417517	14	rs3850378	C	T	NA	0.001378	55.54719
0.0187	40266	2.26E-19	0.1684	67441750	15	rs72743461	A	C	NA	0.00201	81.09226
0.0182	40266	2.63E-09	-0.1082	11344903	16	rs2021511	T	C	NA	0.000877	35.34192
0.0167	40266	8.87E-14	0.1243	28494421	16	rs42861	G	A	NA	0.001374	55.39719
0.0174	40266	1.8E-108	0.385	50756881	16	rs2076756	G	A	NA	0.012013	489.5549
0.0209	40266	2.09E-10	-0.1327	50353529	16	rs7195228	G	C	NA	0.001	40.31138
0.0382	40266	6.05E-47	0.5495	50866917	16	rs72798422	C	T	NA	0.005113	206.9127
0.0176	40266	1.26E-09	0.1067	82867456	16	rs10492862	A	C	NA	0.000912	36.75208
0.0163	40266	5.15E-10	0.1016	25856486	17	rs2948542	G	A	NA	0.000964	38.84997
0.0181	40266	2.49E-17	-0.1531	32617265	17	rs714910	C	A	NA	0.001774	71.54374
0.016	40266	4.31E-19	0.1426	38043649	17	rs12936409	T	C	NA	0.001969	79.42871

0.0162	40266	1.8E-12	-0.1142	40514201	17	rs744166	G	A	NA	0.001233	49.69133
0.0244	40266	1.34E-20	0.2268	12818922	18	rs80262450	A	G	NA	0.002141	86.39426
0.047	40266	2.69E-15	-0.3712	10492274	19	rs144309607	T	C	NA	0.001547	62.37329
0.0201	40266	8.61E-13	0.144	33753200	19	rs62126620	A	G	NA	0.001273	51.32291
0.0193	40266	6.03E-21	0.1811	1123652	19	rs4807570	A	G	NA	0.002182	88.04419
0.0165	40266	2.65E-11	-0.1098	44634912	20	rs3761158	A	G	NA	0.001099	44.28078
0.0163	40266	1.59E-27	-0.1769	16816017	21	rs1297264	G	A	NA	0.002917	117.7766
0.0165	40266	1.14E-14	0.1277	34776695	21	rs2284553	G	A	NA	0.001485	59.89524
0.0162	40266	2.03E-19	-0.1456	45613825	21	rs2838517	C	T	NA	0.002002	80.77392
0.0223	40266	6.84E-21	-0.2087	39660829	22	rs2143178	C	T	NA	0.00217	87.58175
0.0206	40266	3.02E-16	0.1687	21916166	22	rs5754100	C	T	NA	0.001663	67.06164

Table S5. 60 index SNPs represented genetically predicted UC.

se.exposure	beta.exposure	chr.exposure	pos.exposure	pval.exposure	samplesize.exposure	SNP	effect_allele.exposure	other_allele.exposure	eaf.exposure	R2	F-statistic
0.016	-0.1168	1	2496649	2.53E-13	45975	rs7544646	G	C	NA	0.001158	53.28768
0.0209	0.21	1	2.07E+08	7.46E-24	45975	rs3024493	A	C	NA	0.002191	100.9548
0.0178	-0.1587	1	20142413	3.91E-19	45975	rs3820330	A	C	NA	0.001726	79.48679
0.0358	-0.483	1	67705958	1.99E-41	45975	rs11209026	A	G	NA	0.003944	182.0159
0.016	-0.1569	1	1.61E+08	1.17E-22	45975	rs6658353	C	G	NA	0.002087	96.15836
0.0178	-0.1448	1	2.01E+08	4.27E-16	45975	rs7554511	A	C	NA	0.001437	66.1726
0.021	-0.1389	1	8180210	3.42E-11	45975	rs7523335	A	G	NA	0.000951	43.74687
0.0229	0.1375	1	2E+08	1.8E-09	45975	rs2816954	A	T	NA	0.000784	36.05085
0.0203	0.1818	1	20140036	2.9E-19	45975	rs1317209	A	G	NA	0.001741	80.20045
0.0264	0.1605	1	1.52E+08	1.3E-09	45975	rs79051659	A	G	NA	0.000803	36.95927
0.0159	-0.2217	1	20227723	2.61E-44	45975	rs4654925	C	G	NA	0.004211	194.4098
0.0161	0.1597	2	61204641	3.03E-23	45975	rs7608697	C	A	NA	0.002136	98.38733
0.0166	0.1054	2	2E+08	2.09E-10	45975	rs55905347	A	G	NA	0.000876	40.3131
0.0171	0.1226	2	2E+08	8.08E-13	45975	rs62180181	T	C	NA	0.001117	51.40079
0.0167	0.1433	2	2.42E+08	1.19E-17	45975	rs4676408	A	G	NA	0.001599	73.62758
0.0223	-0.1299	2	2.29E+08	6.09E-09	45975	rs1811711	G	C	NA	0.000738	33.93045
0.0168	0.1593	3	49714225	2.18E-21	45975	rs1131095	C	T	NA	0.001952	89.90712
0.0171	0.1714	5	1.59E+08	9.73E-24	45975	rs755374	T	C	NA	0.002181	100.464
0.0206	-0.1223	5	554211	2.89E-09	45975	rs72704802	T	C	NA	0.000766	35.24517
0.0166	0.0974	5	1.34E+08	4.62E-09	45975	rs17715902	A	G	NA	0.000748	34.42571
0.0228	0.1318	5	40347469	7.87E-09	45975	rs6889364	A	G	NA	0.000726	33.41506

0.0159	0.09	5	1.5E+08	1.54E-08	45975	rs17656349	T	C	NA	0.000696	32.03848
0.0171	0.0944	5	1.77E+08	3.27E-08	45975	rs67111717	G	A	NA	0.000662	30.47424
0.0184	-0.1102	6	29941617	1.96E-09	45975	rs9260809	G	A	NA	0.00078	35.86812
0.028	0.2486	6	32044834	6.54E-19	45975	rs9267798	C	G	NA	0.001712	78.8256
0.0165	-0.1468	6	32583653	4.65E-19	45975	rs28383224	G	A	NA	0.001719	79.15263
0.0584	0.5033	6	31030303	6.58E-18	45975	rs3734851	A	G	NA	0.001613	74.26929
0.0436	0.2944	6	1.12E+08	1.48E-11	45975	rs13200059	A	G	NA	0.000991	45.59149
0.0188	0.1486	6	1.38E+08	2.69E-15	45975	rs6933404	C	T	NA	0.001357	62.47453
0.0531	-0.3066	6	19781009	7.59E-09	45975	rs113986290	T	C	NA	0.000725	33.33776
0.0179	-0.1206	7	2788912	1.47E-11	45975	rs798506	C	T	NA	0.000986	45.39099
0.0158	0.0995	7	1.29E+08	3.23E-10	45975	rs4728142	A	G	NA	0.000862	39.65638
0.016	-0.1214	7	1.07E+08	3.28E-14	45975	rs989960	T	C	NA	0.001251	57.56765
0.016	-0.1512	7	1.07E+08	4.11E-21	45975	rs10272963	T	C	NA	0.001939	89.29862
0.0166	-0.167	9	4984530	9.65E-24	45975	rs1887428	C	G	NA	0.002197	101.204
0.017	0.1332	9	1.18E+08	4.42E-15	45975	rs10817678	A	G	NA	0.001334	61.38916
0.016	0.1335	9	1.39E+08	6.5E-17	45975	rs3812565	C	T	NA	0.001512	69.61514
0.016	0.1276	10	64445564	1.33E-15	45975	rs10761659	G	A	NA	0.001381	63.59786
0.0239	-0.1342	10	27179596	1.84E-08	45975	rs7911117	G	T	NA	0.000685	31.52756
0.0159	-0.1525	10	1.01E+08	6.71E-22	45975	rs7911680	C	A	NA	0.001997	91.98702
0.0159	0.1252	11	76281593	2.8E-15	45975	rs2212434	T	C	NA	0.001347	62.00055
0.0169	-0.1063	11	1.14E+08	2.83E-10	45975	rs2045241	A	G	NA	0.00086	39.56163
0.0161	0.1889	12	68492980	7.33E-32	45975	rs12825700	A	G	NA	0.002985	137.6554
0.0202	0.1571	13	27536972	6.58E-15	45975	rs1359946	A	G	NA	0.001314	60.48264
0.0184	0.1078	15	67455630	4.66E-09	45975	rs56062135	T	C	NA	0.000746	34.32282
0.02	-0.1174	16	23847062	4.14E-09	45975	rs11645239	G	C	NA	0.000749	34.4554

0.0189	0.1071	16	68587692	1.41E-08	45975	rs7203363	A	T	NA	0.000698	32.10971
0.0214	0.1357	16	86009740	2.18E-10	45975	rs16940186	C	T	NA	0.000874	40.20807
0.0229	-0.1627	17	70611194	1.15E-12	45975	rs113846785	CG	C	NA	0.001097	50.47604
0.0158	0.1365	17	38043649	5.62E-18	45975	rs12936409	T	C	NA	0.001621	74.63323
0.0219	0.147	17	40759937	2.01E-11	45975	rs11651246	G	T	NA	0.000979	45.0534
0.0204	0.1548	19	33754044	2.92E-14	45975	rs10408351	A	G	NA	0.001251	57.57881
0.0308	0.1759	19	10562802	1.08E-08	45975	rs78064630	A	G	NA	0.000709	32.61455
0.017	0.1944	20	43065028	3.95E-30	45975	rs6017342	C	A	NA	0.002836	130.7603
0.0186	-0.2217	21	40466299	1.11E-32	45975	rs2836881	T	G	NA	0.003081	142.0648
0.016	-0.1177	21	45613825	1.78E-13	45975	rs2838517	C	T	NA	0.001176	54.11206
0.0161	-0.1227	21	16833222	2.22E-14	45975	rs1736161	A	G	NA	0.001262	58.07891
0.0227	-0.1494	22	39662480	5.11E-11	45975	rs9611131	C	T	NA	0.000941	43.31423
0.0179	-0.0988	22	30253256	3.54E-08	45975	rs4993442	T	G	NA	0.000662	30.46414
0.0158	0.1011	22	50439430	1.5E-10	45975	rs137845	G	A	NA	0.00089	40.94202

Table S6. 13 index SNPs represented genetically predicted IL-6 levels.

chr.exposure	pos.exposure	beta.exposure	se.exposure	pval.exposure	samplesize.exposure	SNP	effect_allele.exposure	other_allele.exposure	eaf.exposure	R2	F-statistic
1	1.82E+08	0.4099	0.0893	4.44E-06	21758	rs111572787	T	C	0.0109	0.003623	79.10571
1	1.54E+08	0.1747	0.0124	3.34E-45	21758	rs2228145	C	A	0.3786	0.01436	316.9776
1	43014917	-0.1821	0.0379	1.51E-06	21758	rs148924545	G	A	0.0376	0.0024	52.33785
2	1.55E+08	-0.0573	0.0122	2.69E-06	21758	rs1444691	G	A	0.402	0.001579	34.39788
3	1.64E+08	-0.0679	0.0147	4.11E-06	21758	rs9811834	T	A	0.4542	0.002286	49.84518
3	1.08E+08	-0.1956	0.0418	2.92E-06	21758	rs80316479	T	C	0.027	0.00201	43.82251
4	55033811	-0.4095	0.0884	3.57E-06	21758	rs34032964	T	A	0.0127	0.004205	91.87553
5	1.59E+08	0.0631	0.0128	8.68E-07	21758	rs7448500	G	C	0.694	0.001691	36.85392
7	1.4E+08	-0.2511	0.0506	7.01E-07	21758	rs76598619	C	T	0.0237	0.002918	63.66535
11	1.22E+08	-0.3623	0.0731	7.16E-07	21758	rs144530765	G	A	0.0126	0.003266	71.29025
16	4439131	0.068	0.0147	3.95E-06	21758	rs1530088	T	C	0.7709	0.001633	35.59262
19	30102856	-0.0617	0.0133	3.74E-06	21758	rs2288477	G	A	0.3368	0.001701	37.06254
19	35481231	-0.071	0.0155	4.52E-06	21758	rs2651097	A	G	0.2506	0.001893	41.27086

Table S7. 7 index SNPs represented genetically predicted IL-6α.

chr.exposure	pos.exposure	beta.exposure	se.exposure	pval.exposure	samplesize.exposure	SNP	effect_allele.exposure	other_allele.exposure	eaf.exposure	R2	F-statistic
1	1.54E+08	0.3009	0.0321	6.62E-21	21758	rs61805738	A	G	0.0363	0.006335	138.6953
1	1.54E+08	0.3534	0.0128	1.2E-168	21758	rs72633650	C	T	0.138	0.029713	666.2364
1	1.55E+08	0.452	0.0123	1E-200	21758	rs11264224	C	A	0.181	0.060572	1402.764
1	1.53E+08	0.1956	0.0279	2.42E-12	21758	rs72702900	A	T	0.0396	0.00291	63.49796
1	1.54E+08	-0.5735	0.0395	9.27E-48	21758	rs116141616	A	G	0.0274	0.01753	388.1874
1	1.55E+08	-0.2534	0.012	1.19E-98	21758	rs61811421	T	C	0.2443	0.023709	528.343
1	1.55E+08	-0.0577	0.0103	2.21E-08	21758	rs1218552	A	G	0.6463	0.001522	33.16587

Table S8. Information of outliers detected by MR PRESSO.

MR PRESSO: NbDistribution = 3000			
**exposure**	**outcome**	**structure**	**outliers (SNP)**
IBD	Global	TH	rs11236797
IBD	lateralorbitofrontal	SA	rs62378712
			rs62408218
IBD	parstriangularis	SA	rs6584282
IBD	transversetemporal	SA	rs6873866
IBD	cuneus	TH	rs80262450
IBD	insula	TH	rs111456533
			rs11734570
IBD	isthmuscingulate	TH	rs111456533
IBD	medialorbitofrontal	TH	rs11236797
CD	lingual	SA	rs12131079
CD	pericalcarine	SA	rs42861
CD	superiorparietal	SA	rs3761158
			rs5754100
CD	temporalpole	SA	rs73243877
CD	transversetemporal	SA	rs2188962
			rs6873866
CD	cuneus	TH	rs80262450
CD	isthmuscingulate	TH	rs2188962
CD	superiorfrontal	TH	rs42861

UC	global	TH	rs13200059
			rs2212434
UC	entorhinal	SA	rs7554511
UC	superiorfrontal	SA	rs7554511
UC	superiortemporal	SA	rs7523335
UC	parahippocampal	TH	rs7554511
IL-6	lateralorbitofrontal	SA	rs7448500
IL-6	pericalcarine	SA	rs148924545
IL-6	caudalmiddlefrontal	TH	rs111572787
IL-6	precentral	TH	rs1444691
			rs7448500

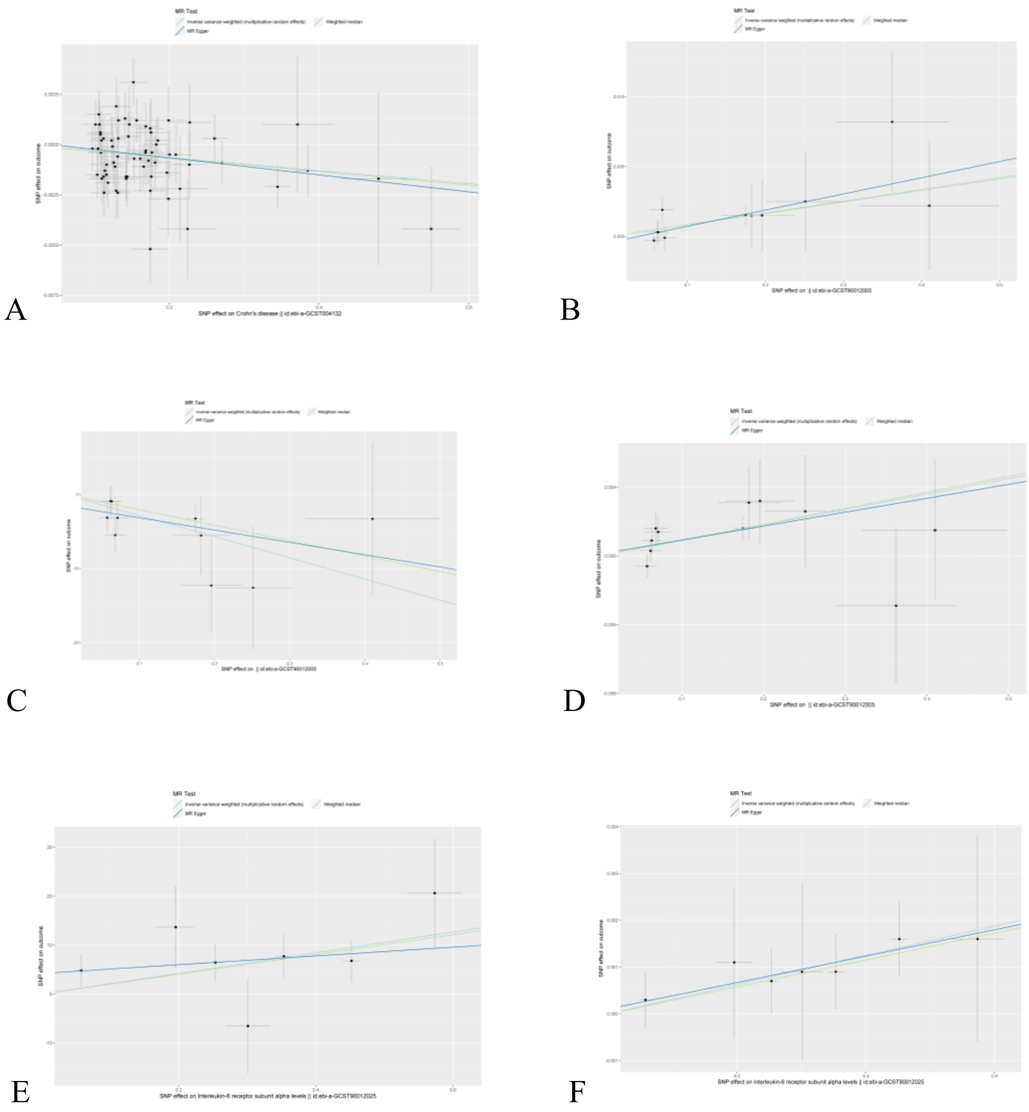

Figure S1. Scatter plots of significant results from MR analysis. (A) genetically predicted CD on TH of pars orbitalis; (B) genetically predicted IL-6 on TH of fusiform; (C) genetically predicted IL-6 on SA of middle temporal; (D) genetically predicted IL-6 on TH of pars opercularis; (E) genetically predicted IL-6Rα on SA of superior frontal; (F) genetically predicted IL-6Rα on TH of supramarginal.

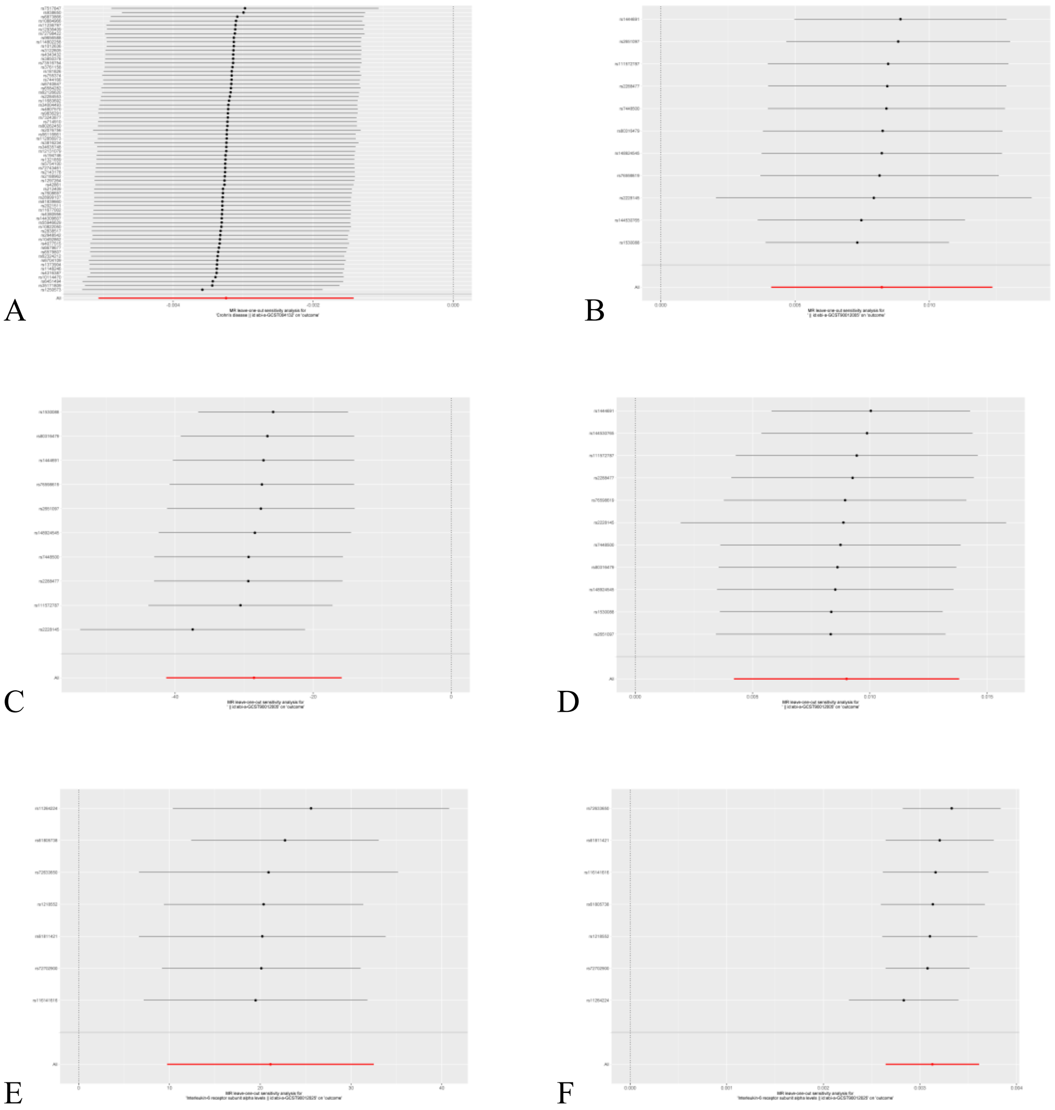

Figure S2. Leave-one-out analysis of significant results from MR analysis. (A) genetically predicted CD on TH of pars orbitalis; (B) genetically predicted IL-6 on TH of fusiform; (C) genetically predicted IL-6 on SA of middle temporal; (D) genetically predicted IL-6 on TH of pars opercularis; (E) genetically predicted IL-6Rα on SA of superior frontal; (F) genetically predicted IL-6Rα on TH of supramarginal.

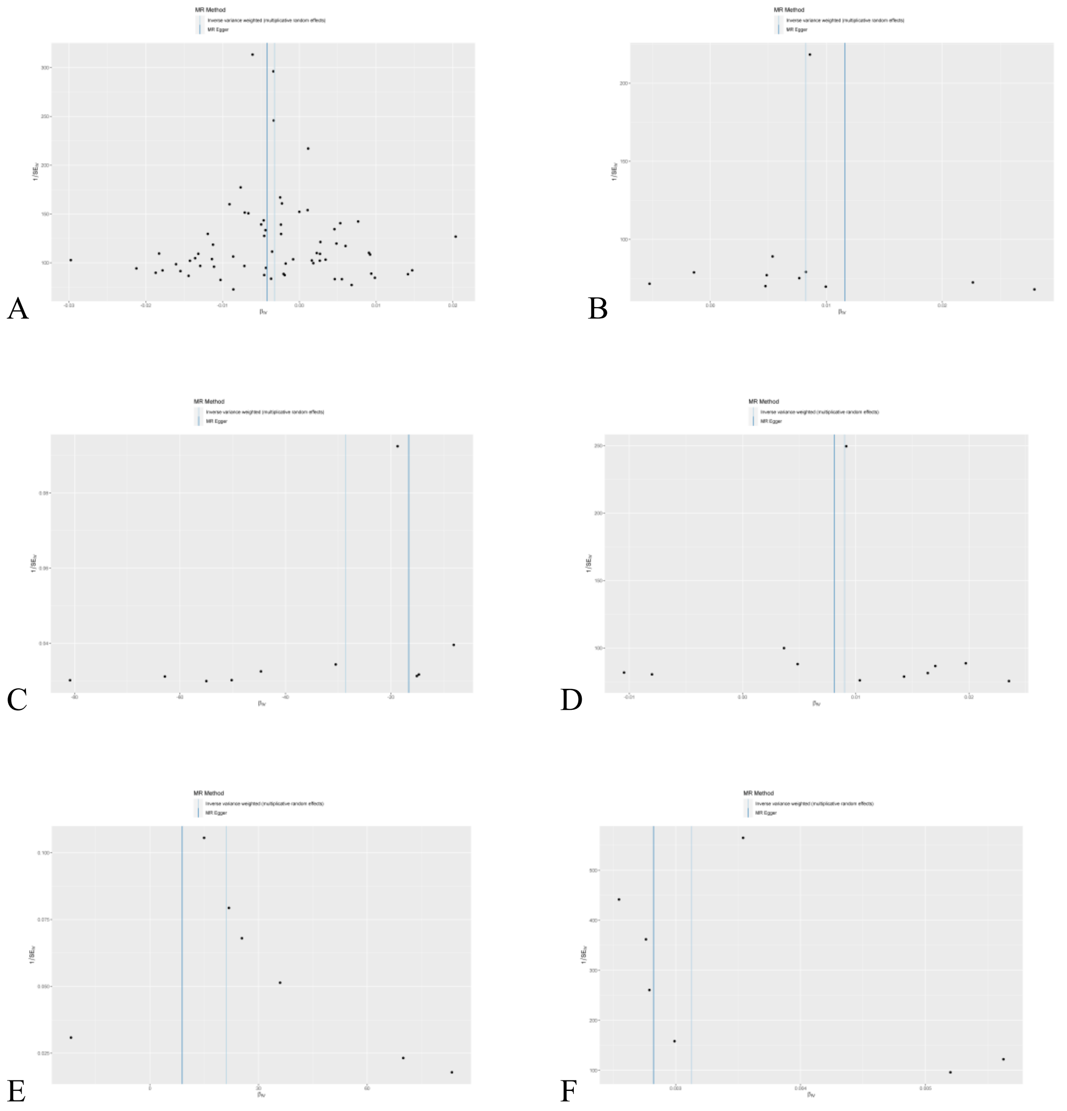

Figure S3. Funnel plots of significant results from MR analysis. (A) genetically predicted CD on TH of pars orbitalis; (B) genetically predicted IL-6 on TH of fusiform; (C) genetically predicted IL-6 on SA of middle temporal; (D) genetically predicted IL-6 on TH of pars opercularis; (E) genetically predicted IL-6Rα on SA of superior frontal; (F) genetically predicted IL-6Rα on TH of supramarginal.

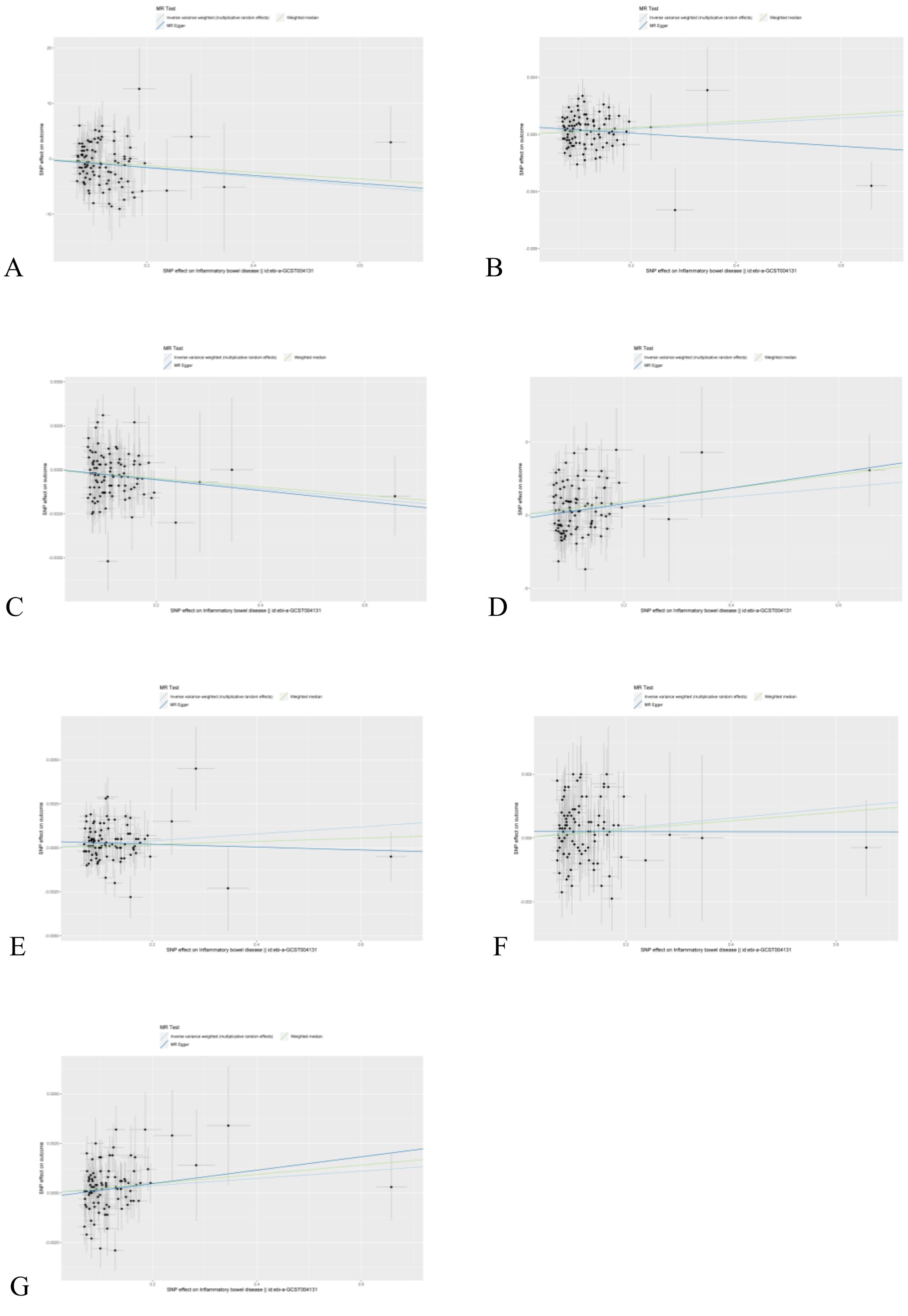

Figure S4. Scatter plots of nominal significant results from genetically predicted IBD on brain cortical structures. (A) SA of inferior parietal; (B) TH of paracentral; (C) TH of pars orbitalis; (D) SA of pars triangularis; (E) TH of postcentral; (F) TH of precentral; (G) TH of cuneus.

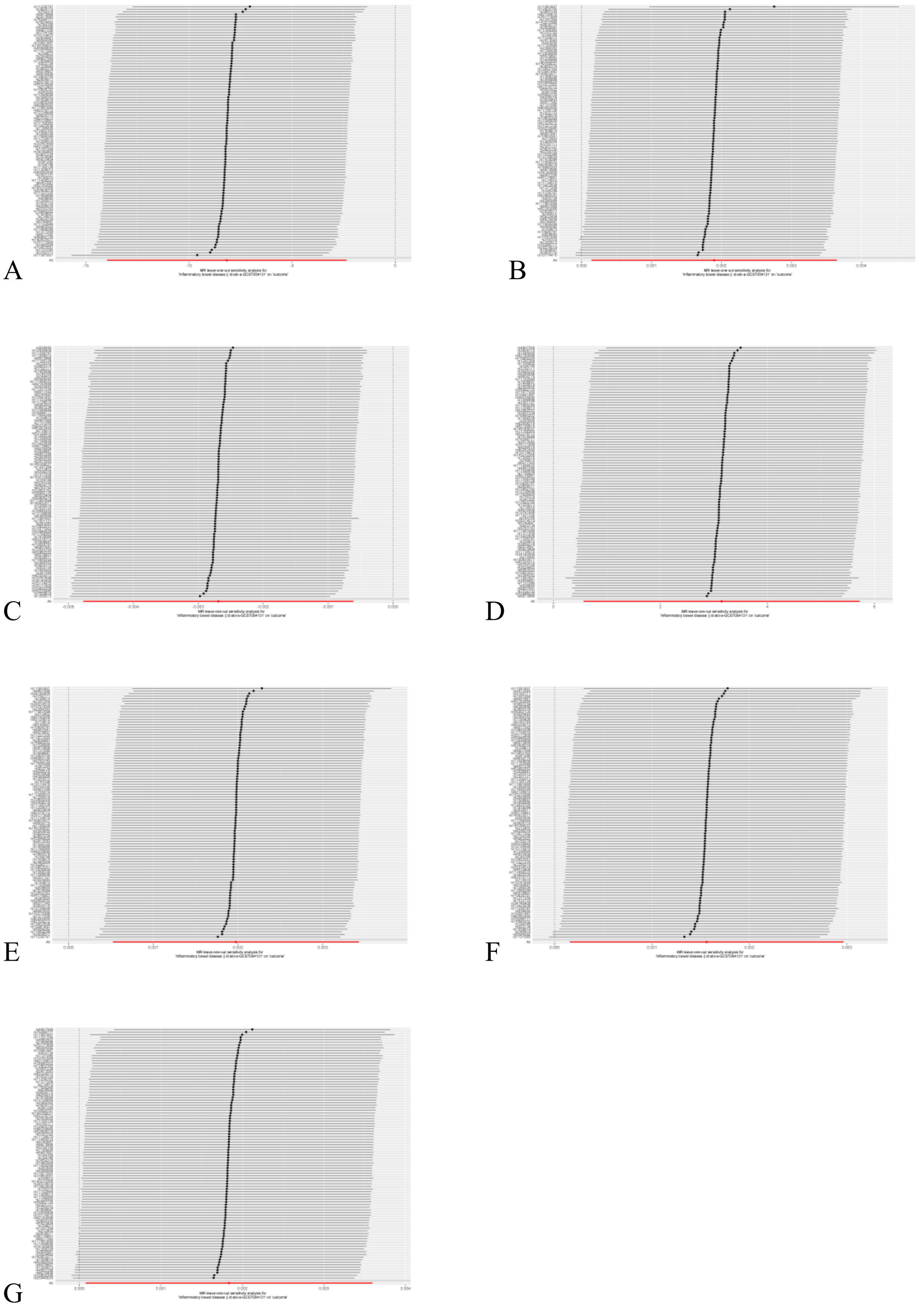

Figure S5. Leave-one-out analysis of nominal significant results from genetically predicted IBD on brain cortical structures. (A) SA of inferior parietal; (B) TH of paracentral; (C) TH of pars orbitalis; (D) SA of pars triangularis; (E) TH of postcentral; (F) TH of precentral. (G) TH of cuneus.

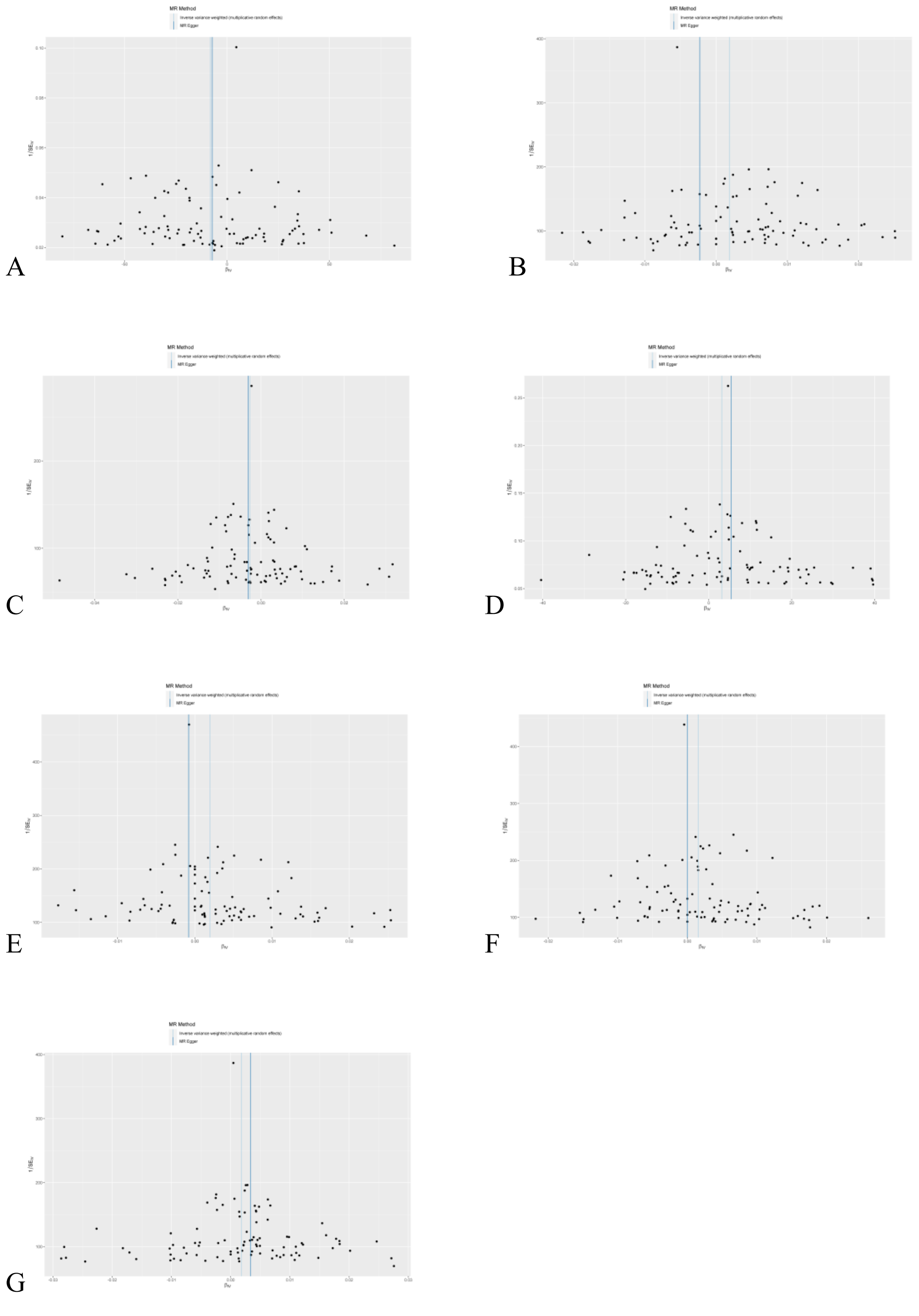

Figure S6. Funnel plots of nominal significant results from genetically predicted IBD on brain cortical structures. (A) SA of inferior parietal; (B) TH of paracentral; (C) TH of pars orbitalis; (D) SA of pars triangularis; (E) TH of postcentral; (F) TH of precentral. (G) TH of cuneus.

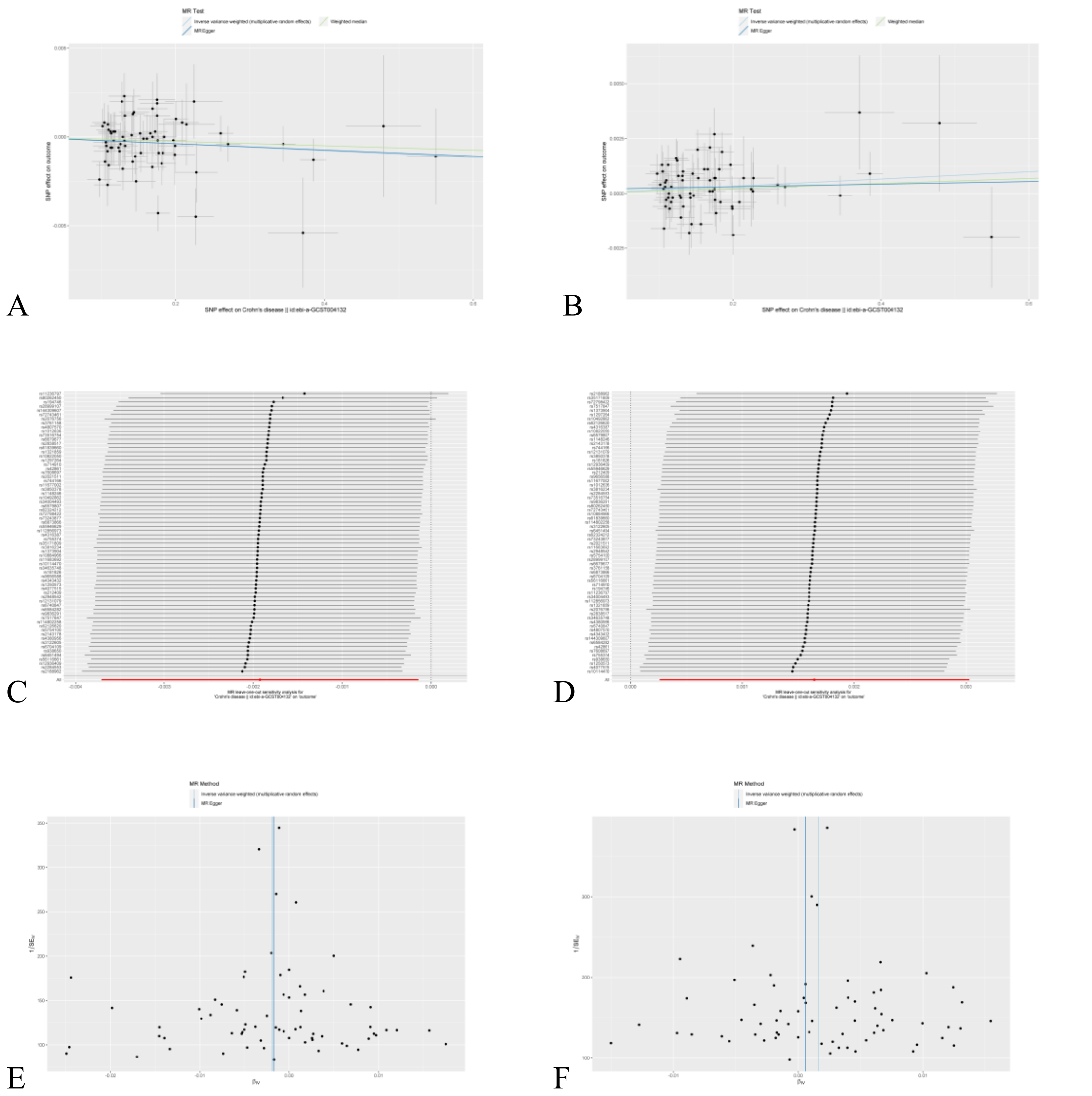

Figure S7. Sensitivity analysis of nominal significant results from genetically predicted CD on brain cortical structures. (A) Scatter plots on TH of medial orbitofrontal; (B) Scatter plots on TH of paracentral; (C) Leave-one-out analysis on TH of medial orbitofrontal; (D) Leave-one-out analysis on TH of paracentral; (E) Funnel plots on TH of medial orbitofrontal; (F) Funnel plots on TH of paracentral.

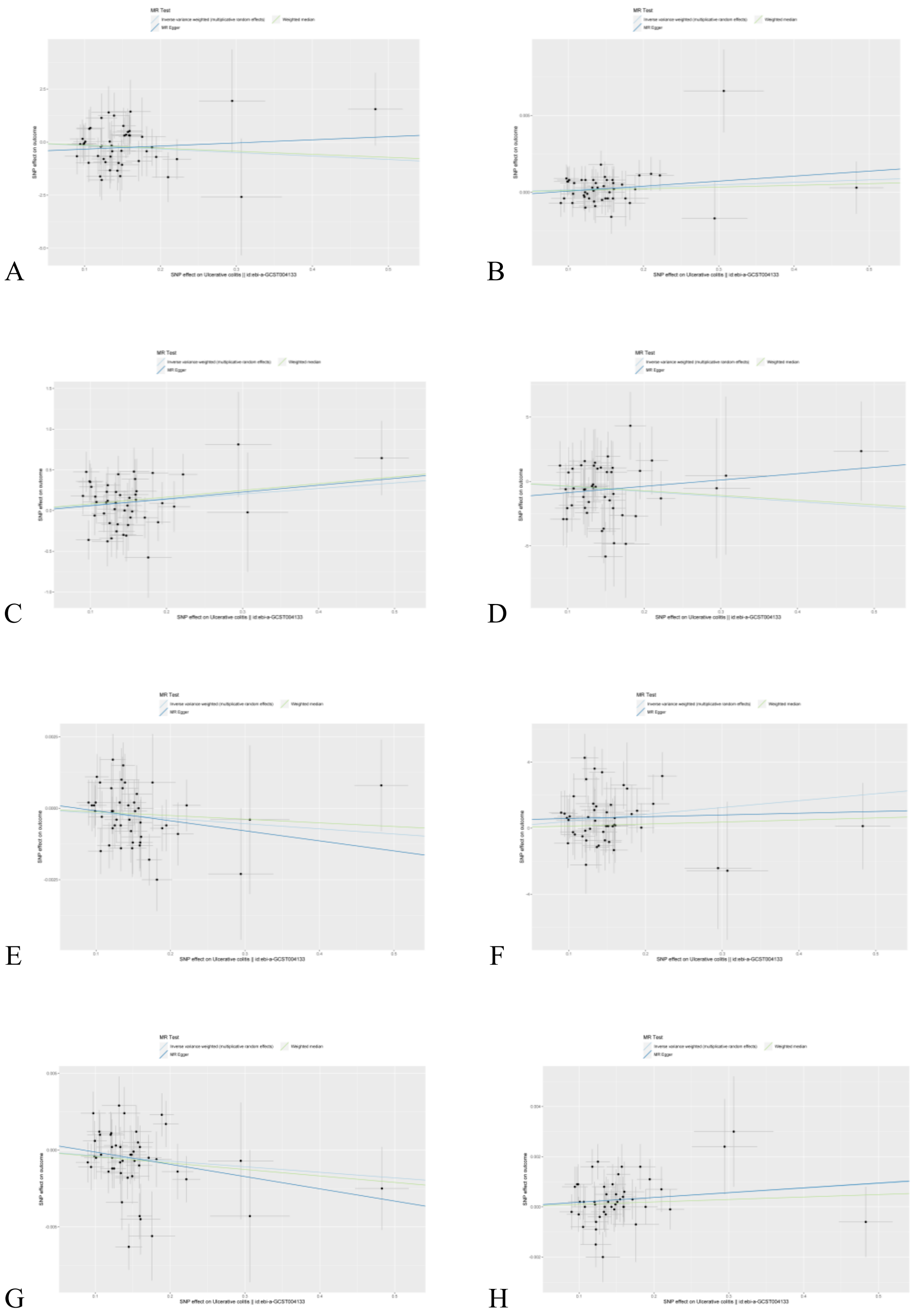

Figure S8. Scatter plots of nominal significant results from genetically predicted UC on brain cortical structures. (A) SA of bankssts; (B) TH of cuneus; (C) SA of frontal pole; (D) SA of fusiform; (E) TH of fusiform; (F) SA of insula; (G) TH of isthmus cingulate; (H) TH of postcentral

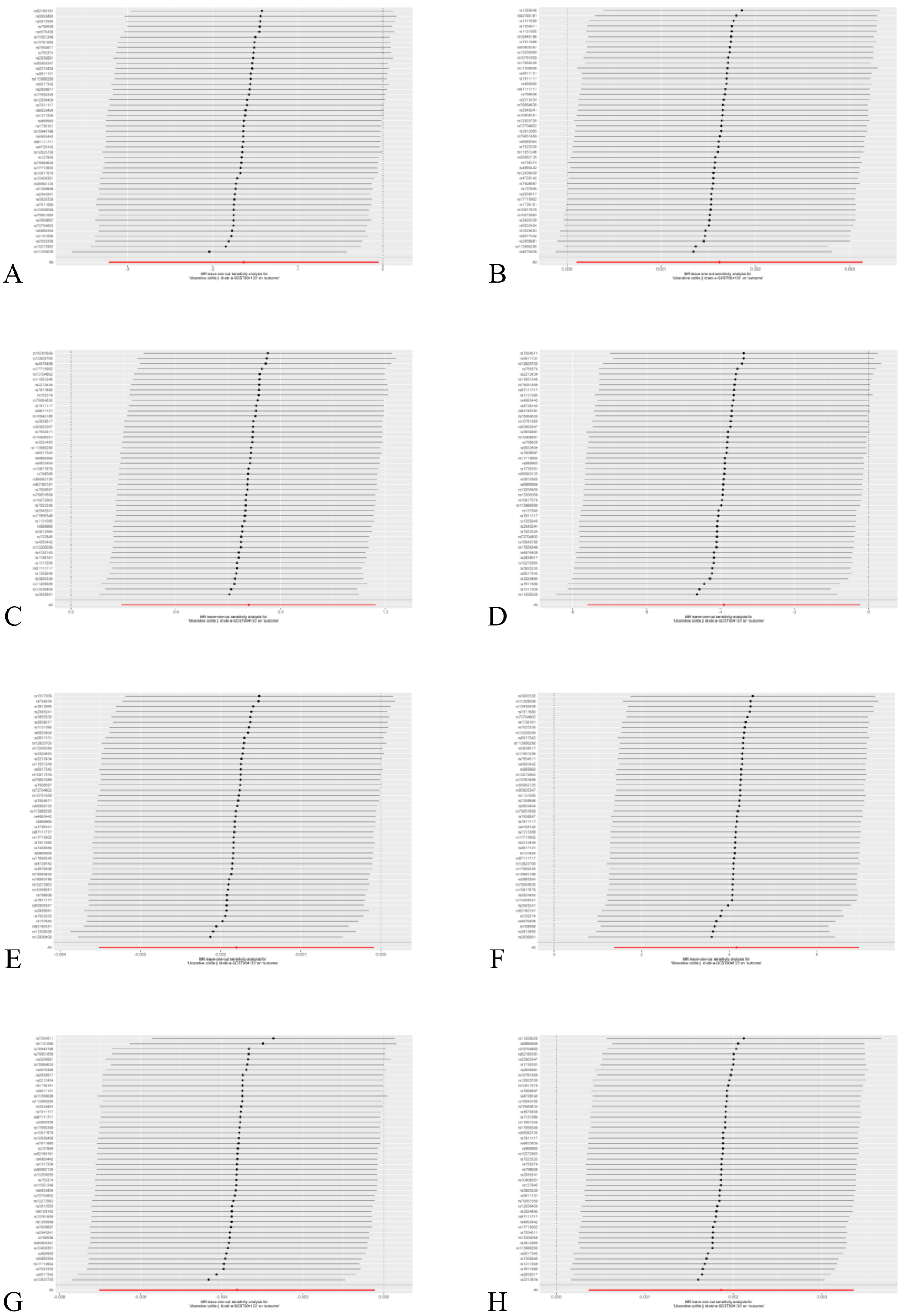

Figure S9. Leave-one-out analysis of nominal significant results from genetically predicted UC on brain cortical structures. (A) SA of bankssts; (B) TH of cuneus; (C) SA of frontal pole; (D) SA of fusiform; (E) TH of fusiform; (F) SA of insula; (G) TH of isthmus cingulate; (H) TH of postcentral.

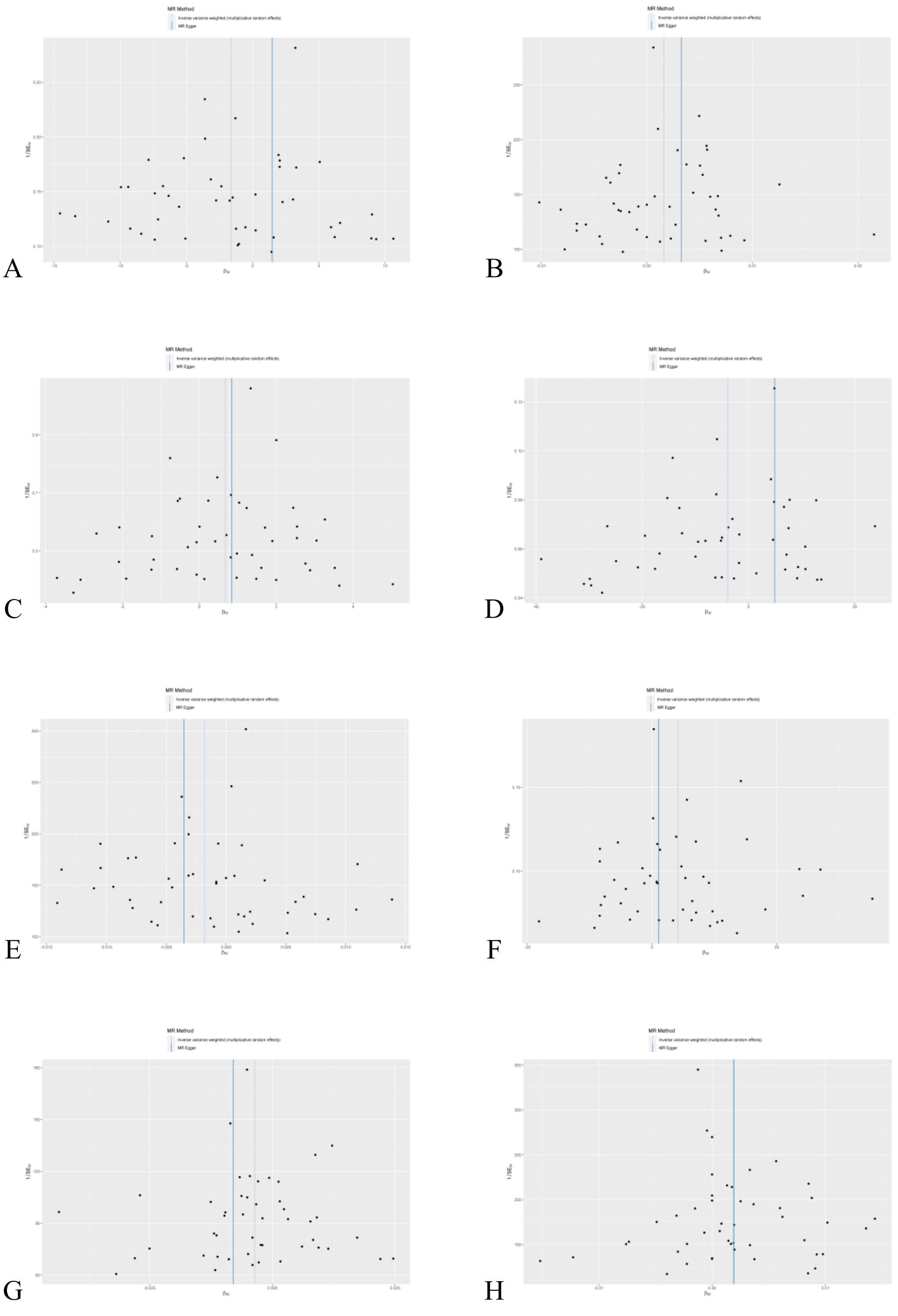

Figure S10. Funnel plots of nominal significant results from genetically predicted UC on brain cortical structures. (A) SA of bankssts; (B) TH of cuneus; (C) SA of frontal pole; (D) SA of fusiform; (E) TH of fusiform; (F) SA of insula; (G) TH of isthmus cingulate; (H) TH of postcentral.

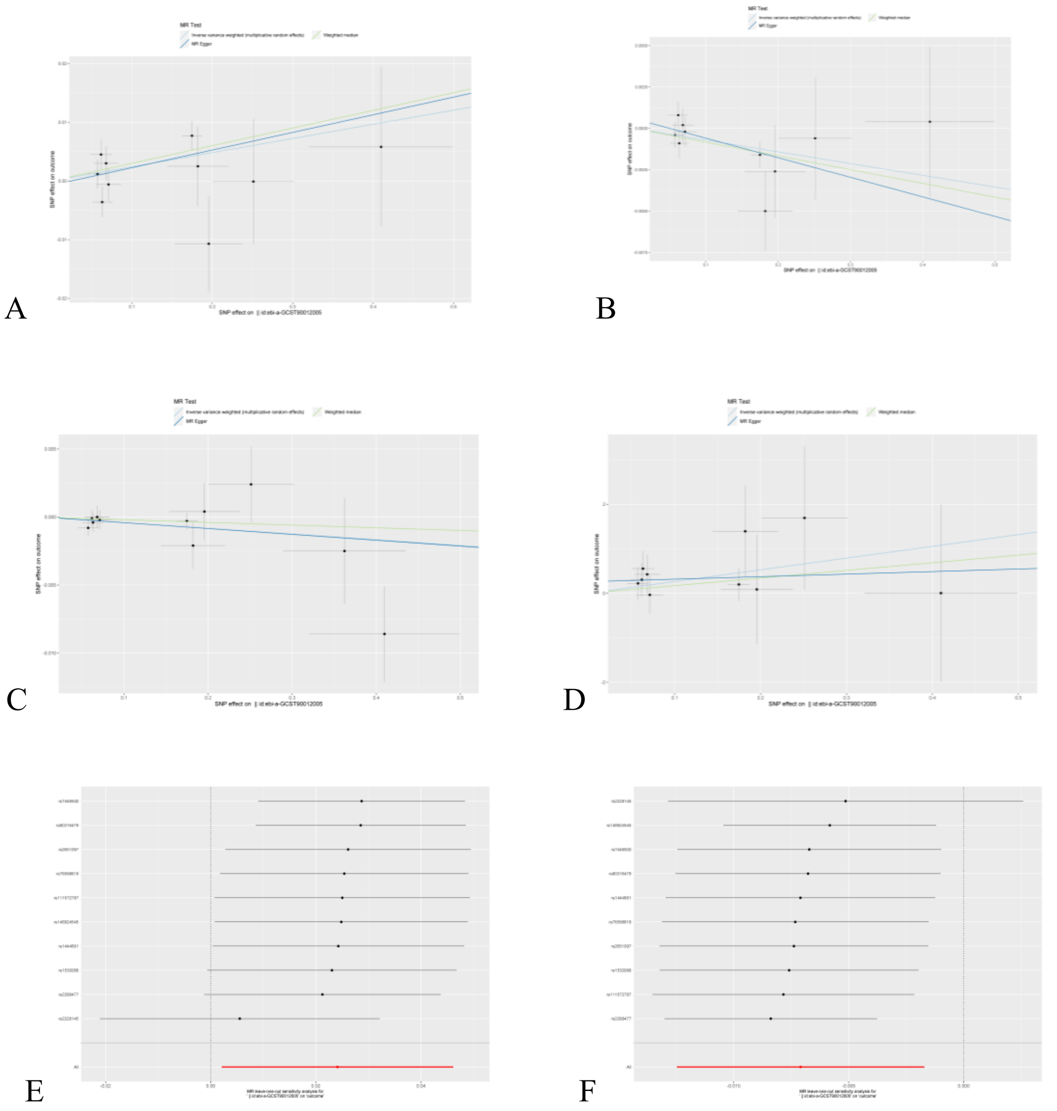

Figure S11. Sensitivity analysis of nominal significant results from genetically predicted IL-6 on brain cortical structures. (A) Scatter plots on TH of entorhinal;(B) Scatter plots on TH of pericalcarine; (C) Scatter plots on TH of superior parietal; (D) Scatter plots on SA of temporal pole; (E)Leave-one-out analysis on TH of entorhinal; (F) Leave-one-out analysis on TH of pericalcarine.

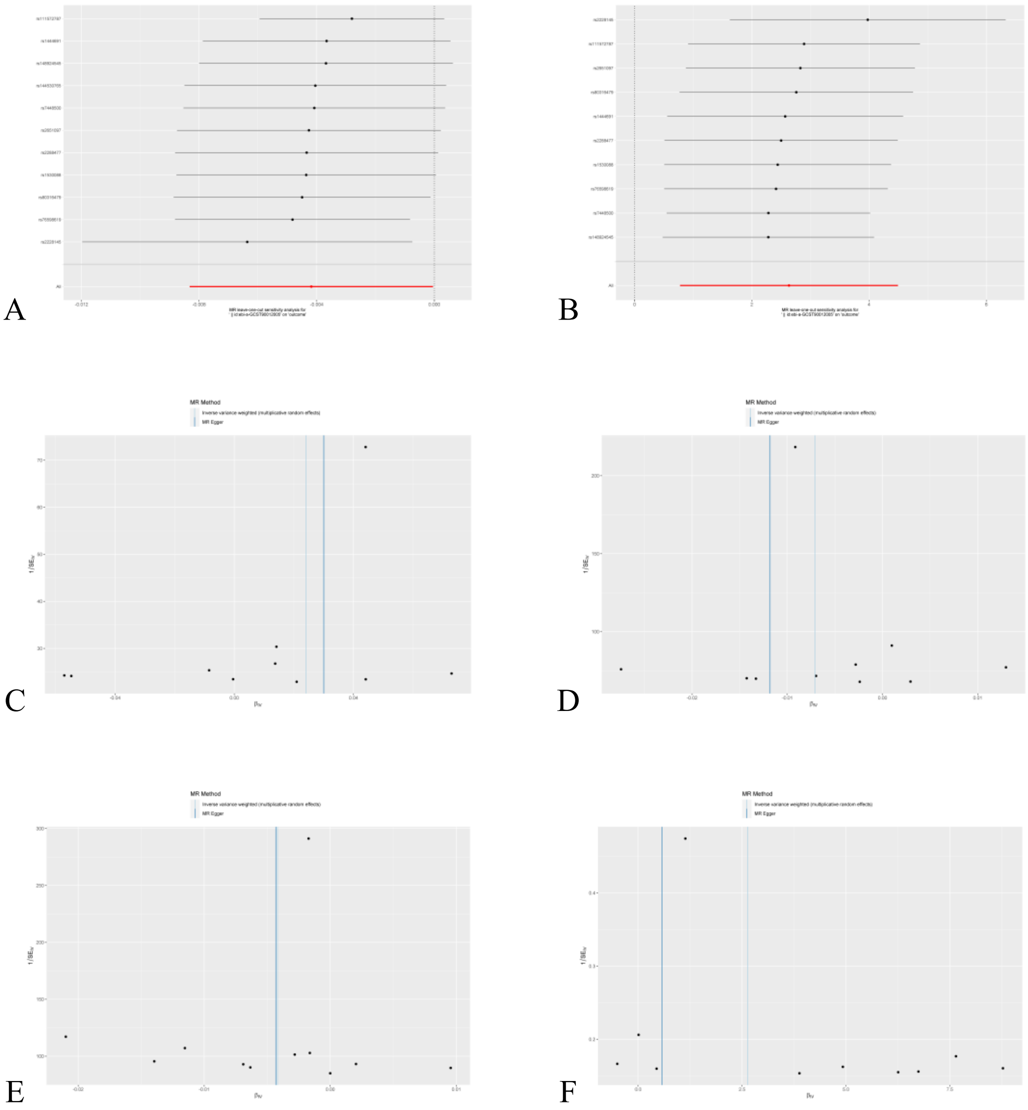

Figure S12. Sensitivity analysis of nominal significant results from genetically predicted IL-6 on brain cortical structures. (A) Leave-one-out analysis on TH of entorhinal;(B) Leave-one-out analysis on TH of pericalcarine; (C) Funnel plots on TH of superior parietal; (D) Funnel plots on SA of temporal pole; (E) Funnel plots on TH of entorhinal; (F) Funnel plots on TH of pericalcarine.

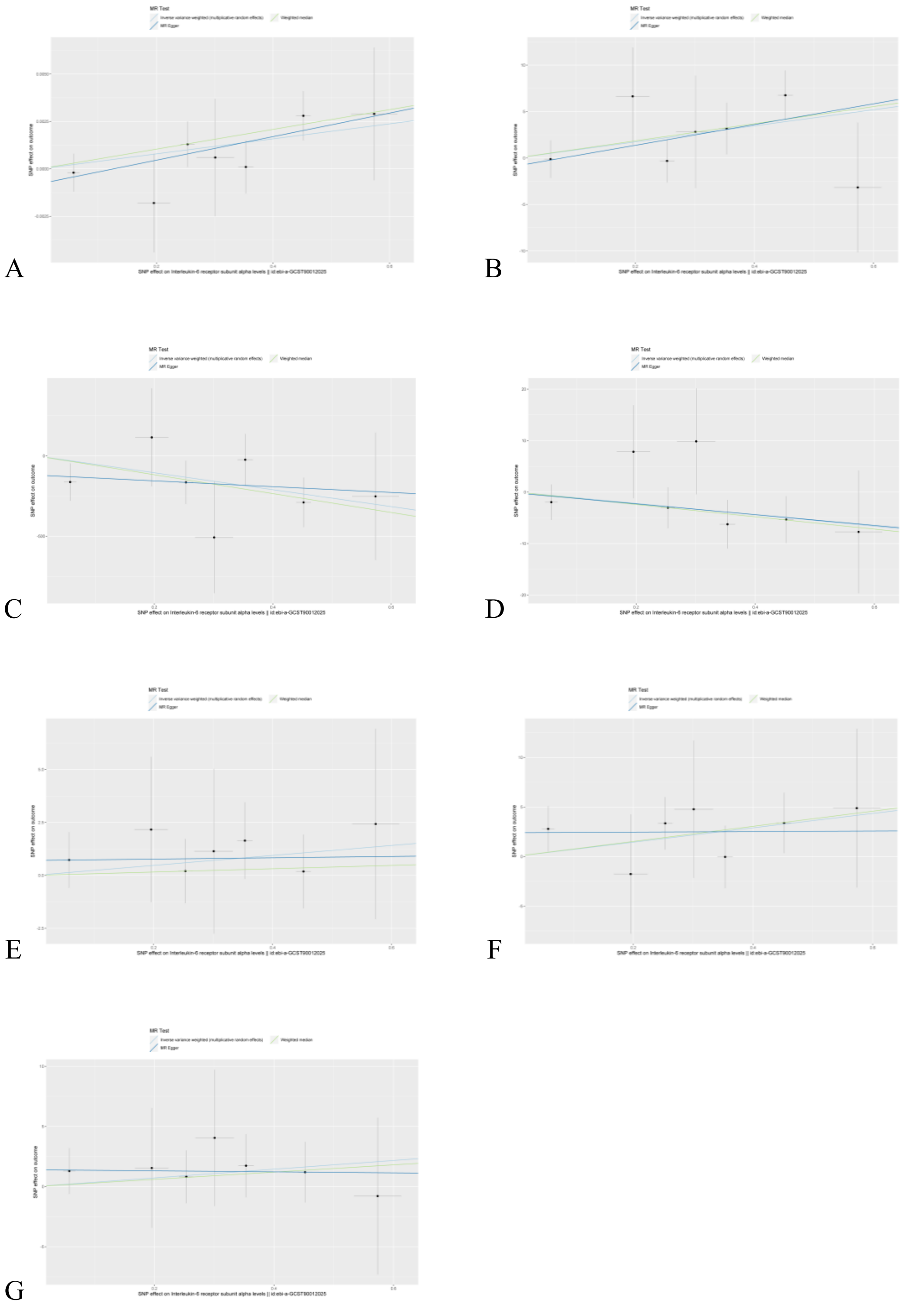

Figure S13. Scatter plots of nominal significant results from genetically predicted IL-6α on brain cortical structures. (A) TH of bankssts; (B) SA of fusiform; (C) SA of Global; (D) SA of inferior parietal; (E) SA of pars triangularis; (F) SA of precuneus; (G) SA of superior temporal.

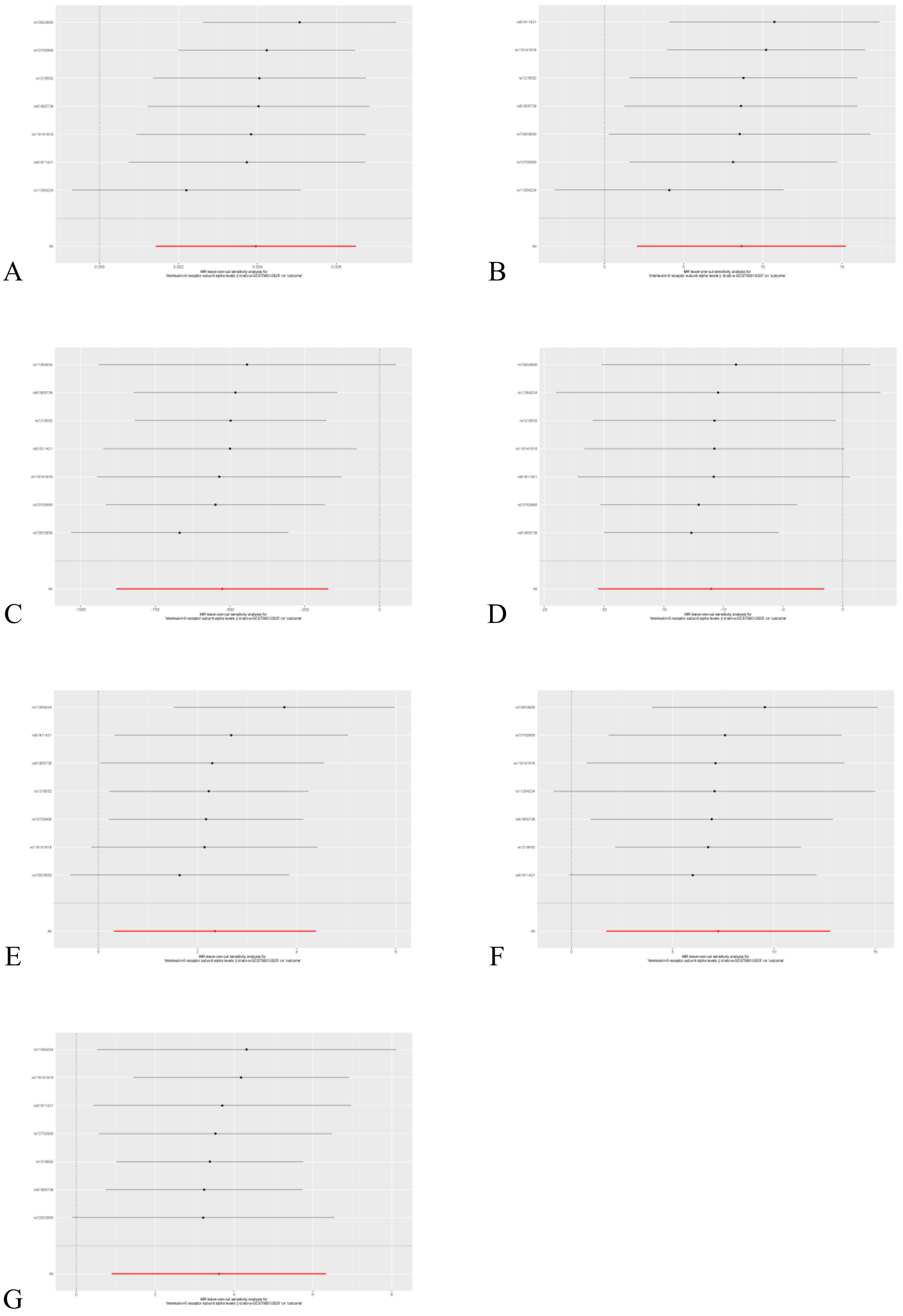

Figure S14. Leave-one-out analysis of nominal significant results from genetically predicted IL-6α on brain cortical structures. (A) TH of bankssts; (B) SA of fusiform; (C) SA of Global; (D) SA of inferior parietal; (E) SA of pars triangularis; (F) SA of precuneus; (G) SA of superior temporal.

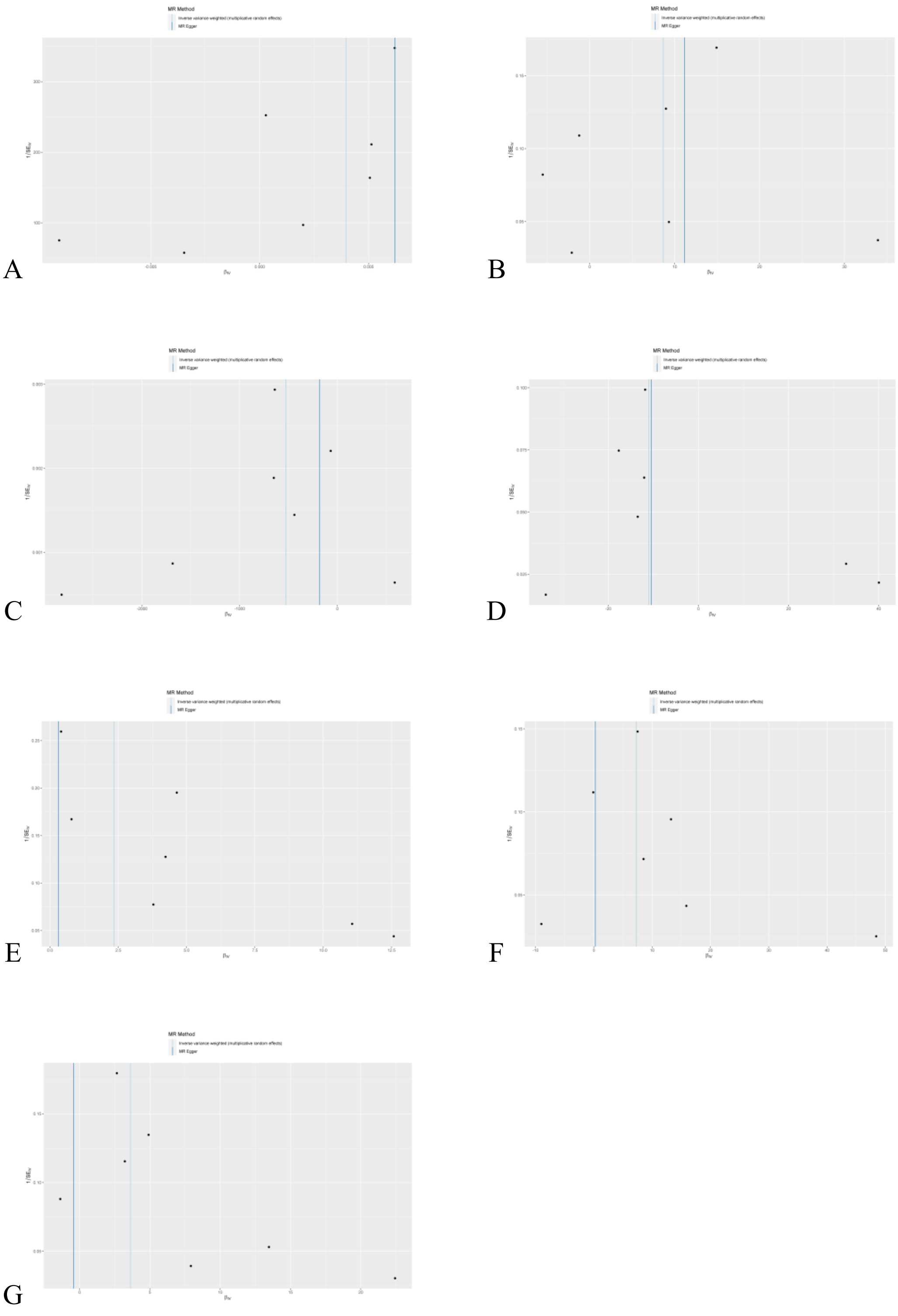

Figure S15. Funnel plots of nominal significant results from genetically predicted IL-6α on brain cortical structures. (A) TH of bankssts; (B) SA of fusiform; (C) SA of Global; (D) SA of inferior parietal; (E) SA of pars triangularis; (F) SA of precuneus; (G) SA of superior temporal.
